# Li iontronics in single-crystalline *T*-Nb_2_O_5_ thin films with vertical ionic transport channels

**DOI:** 10.1038/s41563-023-01612-2

**Published:** 2023-07-27

**Authors:** Hyeon Han, Quentin Jacquet, Zhen Jiang, Farheen N. Sayed, Jae-Chun Jeon, Arpit Sharma, Aaron M. Schankler, Arvin Kakekhani, Holger L. Meyerheim, Jucheol Park, Sang Yeol Nam, Kent J. Griffith, Laura Simonelli, Andrew M. Rappe, Clare P. Grey, Stuart S. P. Parkin

**Affiliations:** 1 Max Planck Institute of Microstructure Physics, Halle (Saale), Germany; 2 Yusuf Hamied Department of Chemistry, University of Cambridge, Cambridge, UK; 3 Department of Chemistry, University of Pennsylvania, Philadelphia, PA USA; 4 Test Analysis Research Center, Gumi Electronics and Information Technology Research Institute, Gumi, Republic of Korea; 5 Department of Chemistry, Northwestern University, Evanston, IL USA; 6 ALBA Synchrotron Light Source, Cerdanyola del Vallès, Barcelona, Spain; 7 Present Address: Univ. Grenoble Alpes, CEA, CNRS, IRIG, SyMMES, Grenoble, France

**Keywords:** Phase transitions and critical phenomena, Electronic materials, Electronic devices

## Abstract

The niobium oxide polymorph *T*-Nb_2_O_5_ has been extensively investigated in its bulk form especially for applications in fast-charging batteries and electrochemical (pseudo)capacitors. Its crystal structure, which has two-dimensional (2D) layers with very low steric hindrance, allows for fast Li-ion migration. However, since its discovery in 1941, the growth of single-crystalline thin films and its electronic applications have not yet been realized, probably due to its large orthorhombic unit cell along with the existence of many polymorphs. Here we demonstrate the epitaxial growth of single-crystalline *T*-Nb_2_O_5_ thin films, critically with the ionic transport channels oriented perpendicular to the film’s surface. These vertical 2D channels enable fast Li-ion migration, which we show gives rise to a colossal insulator–metal transition, where the resistivity drops by 11 orders of magnitude due to the population of the initially empty Nb 4*d*^0^ states by electrons. Moreover, we reveal multiple unexplored phase transitions with distinct crystal and electronic structures over a wide range of Li-ion concentrations by comprehensive in situ experiments and theoretical calculations, which allow for the reversible and repeatable manipulation of these phases and their distinct electronic properties. This work paves the way for the exploration of novel thin films with ionic channels and their potential applications.

## Abstract (web summary)

The two-dimensional layered crystal structure of niobium oxide polymorph *T*-Nb_2_O_5_ exhibits fast Li-ion diffusion that is promising for energy storage applications. Epitaxial growth of single-crystalline *T*-Nb_2_O_5_ thin films with ionic transport channels oriented perpendicular to the surface are now demonstrated.

## Main

The control of the electronic properties of materials via voltage biasing forms the cornerstone of many electronic applications. A recent trend in the field has been to leverage ionic liquid gating (ILG) of oxides, enabling the electric-field control of insulator-to-metal transitions. These electronic transitions via ILG are typically induced by the electrochemical intercalation of O^2−^ or H^+^ ions^1–13^, which can, however, be sluggish, poorly controlled or involve degradation of the electrolyte^6^. Alternatively, intercalation of Li^+^ ions into oxides can provide fast ion diffusion, and, thereby, is fundamental to diverse applications, ranging from Li-ion batteries^14–19^ and electrochromics^20–22^ to electronic devices^23–30^. WO_3_ is known as one of the best-performing electrochemical materials for Li-ionic gating, owing to the large resistance change (up to approximately seven orders of magnitude), fast response and good endurance^9,10,29^. However, this material and its thin-film form can show a limited voltage operation range due to the conversion reaction that occurs with Li ions^31^. Thus, finding new thin-film systems that exhibit rapid and large changes in properties with high stability via Li-ion intercalation can boost various applications. Moreover, understanding the correlation between structure, electrochemical and electronic properties during Li-ion insertion is needed to realize repeatable manipulation of these properties.

**Fig. 1:**
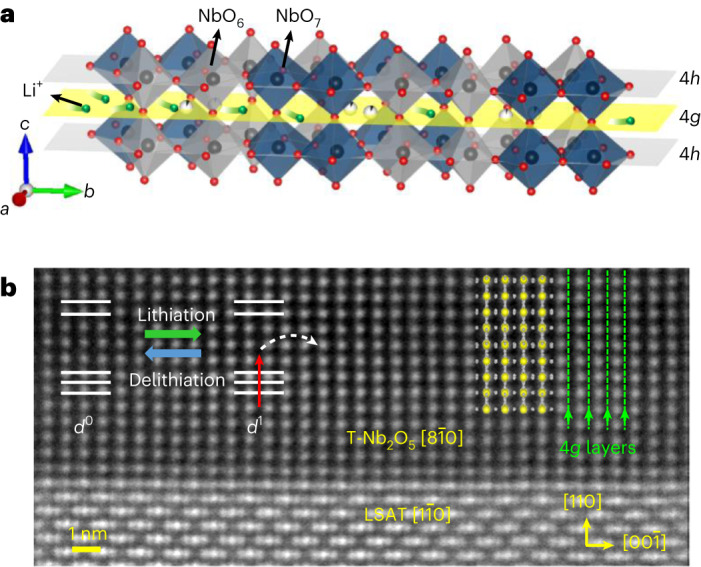
Structure of epitaxial *T*-Nb_2_O_5_ thin films. **a**, Schematic showing Li-ion migration into *T*-Nb_2_O_5_. The black, red and green spheres denote Nb, O and Li ions, respectively. The grey and navy polyhedra denote distorted octahedra (NbO_6_) and pentagonal bipyramids (NbO_7_), respectively. The grey and yellow planes represent the 4*h* and 4*g* layers, respectively. The loosely packed 2D 4*g* layer provides for fast Li-ion transport. **b**, Cross-sectional HAADF-STEM image of a single-crystalline *T*-Nb_2_O_5_ thin-film grown on a LSAT(110) substrate viewed along the LSAT $\left[1\bar{1}0\right]$ direction. Left inset, Nb 4*d* orbital state changes from *d*^0^ to *d*^1^ due to electron doping via Li intercalation. Overlaid yellow and grey spheres represent Nb and O atoms, respectively. Green dashed lines represent the vertical ionic transport channels (4*g* layers) viewed from *T*-Nb_2_O_5_ $[8\bar{1}0]$.

*T*-Nb_2_O_5_ is a promising material, which is known as an anode material for applications in batteries and electrochemical capacitors/pseudocapacitors^32–38^. Fast Li-ion diffusion in *T*-Nb_2_O_5_ is enabled by its crystal structure, consisting of two-dimensional (2D) layers at 4*g* Wyckoff positions of the space group *Pbam*, with very low steric hindrance for intralayer Li-ion diffusion^37,39^ (Fig. 1a and Supplementary Fig. 1). Moreover, *T*-Nb_2_O_5_ is a *d*^0^ insulator, and Li-ion intercalation is expected to increase the electronic conductivity by filling the Nb 4*d* levels (Fig. 1b, left inset), making *T*-Nb_2_O_5_ a promising candidate for switchable electronic applications. However, since the discovery of *T*-Nb_2_O_5_ in 1941^37^, the single-crystalline thin-film growth and its electronic properties have not been demonstrated. This difficulty is probably due to its large orthorhombic unit cell (*a* = 6.175 Å, *b* = 29.175 Å, *c* = 3.930 Å)^39^, its metastability^32^ and the presence of many other Nb_2_O_5_ polymorphs that are close in energy^38^.

Here, we demonstrate epitaxial growth of high-quality single-crystalline *T*-Nb_2_O_5_ thin films oriented with their (180) plane parallel to the surface such that 2D open channels in the crystal structure are ideally oriented perpendicular to the film surface. Li-containing ionic liquid gating (Li-ILG) of *T*-Nb_2_O_5_ thin films results in a fast and colossal insulator–metal transition with approximately 11 orders of magnitude increase in the resistivity of the material during the early stages of Li insertion. Multiple unexplored phase transitions, including an orthorhombic metal and a monoclinic metal, are found in thin-film forms, while the bulk material further reveals a tetragonal phase at high Li concentrations. These new phases were observed by comprehensive in situ experiments and rationalized with the help of density functional theory (DFT) calculations. This understanding enables repeatable and durable control of electronic properties by operating within the reversible phase transition range of the crystalline thin films. Furthermore, a tunable metallization voltage is demonstrated by altering the chemical potential of the gate electrode via the use of lithiated counter electrodes.

## Growth of epitaxial thin films having vertical ionic channels

Pulsed laser deposition (PLD) was used to grow *T*-Nb_2_O_5_ thin films on (001)- and (110)-oriented substrates of LaAlO_3_ (LAO) and (La_0.18_Sr_0.82_)(Al_0.59_Ta_0.41_)O_3_ (LSAT). Various polymorphs, including the TT-, T-, B- and H-Nb_2_O_5_ phases, were identified by X-ray diffraction that strongly depend on the growth temperature (Supplementary Fig. 2a). Single-phase *T*-Nb_2_O_5_(180) films were obtained for growth temperatures between 600 and 650 °C. Cross-sectional scanning transmission electron microscopy (STEM) was used to obtain high-angle annular dark-field (HAADF) images and Fourier transformation patterns (Fig. 1b and Supplementary Fig. 3). When using conventional (001)-oriented substrates, multidomains with the four-fold symmetry, rotated in-plane by 90° with respect to each other, were formed, as previously observed for growth on SrTiO_3_ (001)^40^. However, the growth of two-fold symmetric *T*-Nb_2_O_5_(180) thin films was realized by using (110)-oriented substrates. Reflection high-energy electron diffraction (RHEED) patterns and X-ray diffraction phi-scans further confirmed this finding (Supplementary Section 1). The anisotropic in-plane geometry of the (110)-oriented substrates probably prohibits the formation of multidomains^41^. Both thin films exhibit vertically oriented two-dimensional 4*g* layers (the green dashed lines in Fig. 1b and Supplementary Fig. 3) that are ideal for Li-ion transport.

## Structural and electronic phase diagram by Li insertion

**Fig. 2:**
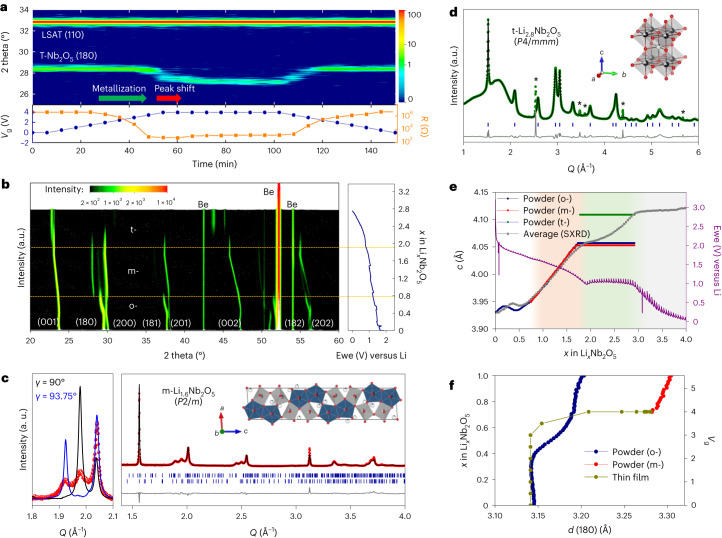
Sequential structural phase transitions of *T*-Nb_2_O_5_ via Li-ion intercalation. **a**, In situ X-ray diffraction and resistance measurements during Li-ILG of a 50 nm *T*-Nb_2_O_5_/LSAT(110). **b**, In situ X-ray diffraction patterns for powder *T*-Nb_2_O_5_ until the deep discharge potential of 0.005 V versus Li in 34 hours. ‘Be’ indicates peaks from the Be window. Horizontal yellow dashed lines indicate the boundary of the transitions. Ewe represents the working electrode potential. **c**, Ex situ SXRD pattern (red) of Li_1.6_Nb_2_O_5_ and simulated patterns of the *Pbam* (black) and *P*2/*m* (blue) structural models. The (180) reflection is split by monoclinic tilting (left panel). SXRD pattern and Rietveld refinements of m-Li_1.6_Nb_2_O_5_. Red circles, black line and grey line represent the observed, calculated and difference patterns, respectively. The black spheres, red spheres, grey polyhedra and navy polyhedra in the unit cell represent Nb ions, O ions, octahedra (NbO_6_) and pentagonal bipyramids (NbO_7_), respectively (right panel). **d**, Rietveld refinement of an in situ SXRD pattern measured at 5 mV using the t-phase. The in situ cell produces peaks marked as *. Green circles, black line and grey line represent the observed, calculated and difference patterns, respectively. Blue vertical bars represent the Bragg position. The black spheres, red spheres and grey polyhedra in the unit cell denote Nb ions, O ions and octahedra (NbO_6_), respectively. Li positions are not considered in all refined structures due to the low scattering amplitude of Li ions. **e**, *c* lattice parameter as a function of *x* in Li_*x*_Nb_2_O_5_. Grey dots represent the average *c* parameter obtained from the SXRD data. Blue, red and green dots denote the *c* parameter of the o*-*, m*-* and t*-*phases, respectively, extracted from the in situ X-ray diffraction data. The purple line is the voltage curve obtained for a powder electrode in a coin cell during a galvanostatic measurement including open circuit voltage steps. Colour zones highlight four regions: (1) pristine *o*-phase from Li_0_Nb_2_O_5_ to Li_0.8_Nb_2_O_5_ (white), (2) a 25/75% mixture of the o- and m-phases from Li_0.8_Nb_2_O_5_ to Li_1.8_Nb_2_O_5_ (pink), (3) progressive formation of the t-phase from Li_1.8_Nb_2_O_5_ to Li_3_Nb_2_O_5_ (green), (4) no change of the X-ray diffraction pattern from Li_3_Nb_2_O_5_ to Li_4_Nb_2_O_5_ (grey). **f**, Comparison of the *d*(180) spacing for the powder and thin-film X-ray diffraction.

The evolution of the crystal structure correlated with corresponding changes of the electronic properties in *T-*Nb_2_O_5_ via Li-ion intercalation was investigated by various in situ and ex situ methods. X-ray diffraction patterns and resistance were measured during Li-ILG of a single-crystalline *T-*Nb_2_O_5_/LSAT(110) thin-film device using Au/Ru as the gate electrode (Fig. 2a and Supplementary Fig. 7). Between a gate voltage (*V*_g_) of 0 and 3 V, there is no noticeable change in the (180) reflection position, while the resistance starts to decrease at *V*_g_ ≈ 2 V, suggesting the onset of an insulator-to-metal transition without a notable structural modification. Beginning with *V*_g_ = 3.5 to 4 V, the (180) diffraction peak starts to shift towards lower angles without any change in resistance; this shift was assigned to a transition from orthorhombic to monoclinic phase by using reciprocal space maps (Supplementary Fig. 8). As shown in Supplementary Fig. 9, in the range *V*_g_ = 4 to –1 V, the (180) peak and resistance return to their original values, indicating the reversibility of the structural and electronic changes. At *V*_g_ = 6 V, concomitantly with the irreversible increase of resistance suggesting a metal-to-insulator transition, an amorphization of the film was observed, evidenced by a decrease of the (180) peak intensity together with TEM images (Supplementary Fig. 10), indicating a degradation of the thin film at high Li concentration.

In situ X-ray diffraction experiments were performed on polycrystalline *T*-Nb_2_O_5_ powder in a typical battery configuration^42^ (Fig. 2b–f and Supplementary Section 2). During Li-ion insertion, a first structural phase transition is revealed at *x* = 0.8 in Li_*x*_Nb_2_O_5_ with a large shift in the (180) peak position along with the disappearance of the (181) peak (Fig. 2b and Supplementary Fig. 11). In addition, at a higher Li-ion content, *x* ≈ 1.8, a second structural transition is observed. To unambiguously identify these unexplored structures, we performed synchrotron-based X-ray diffraction (SXRD) for the Li_0.8_Nb_2_O_5_ and Li_3_Nb_2_O_5_ phases. Rietveld refinement of ex situ SXRD pattern of Li_0.8_Nb_2_O_5_ shows that the transition corresponds to a monoclinic (m-) distortion (*γ* ≈ 94°) of the initial orthorhombic (o-) *T*-Nb_2_O_5_ structure without a notable change in the Nb framework (Fig. 2c, Supplementary Fig. 12 and Supplementary Table 1). Interestingly, ~25% of the crystal volume remains in the orthorhombic phase, while both phases are active for Li intercalation (Supplementary Fig. 15). This suggests that the driving force for the transition to the monoclinic phase of the powder is weak and, hence, in competition with other effects preventing the transition, such as strain or compositional heterogeneities. The second structural transition of the powder involves the formation of a new tetragonal (*t*-) Li-rich layered rock-salt structure with an approximate composition of Li_3_Nb_2_O_5_ (Fig. 2d, Supplementary Fig. 13 and Supplementary Table 2). A recent report mentions the formation of a cubic phase, which can be viewed as a parent phase of this newly discovered tetragonal rock-salt phase, but which is characterized by a disordered Li/Nb site occupancy. Formed by lithiation of an amorphous Nb_2_O_5_ powder, it possesses excellent capacity at higher current densities and with a conductivity that is larger by approximately four orders of magnitude compared to the pristine phase^43^; but we note that this phase still belongs to the insulating regime and is in an irreversible transition range for the initially crystalline thin-film form. Having resolved all phases in the Li_*x*_Nb_2_O_5_, we report the evolution of the cell parameters as a function of Li-ion content together with the voltage (Fig. 2e).

**Fig. 3:**
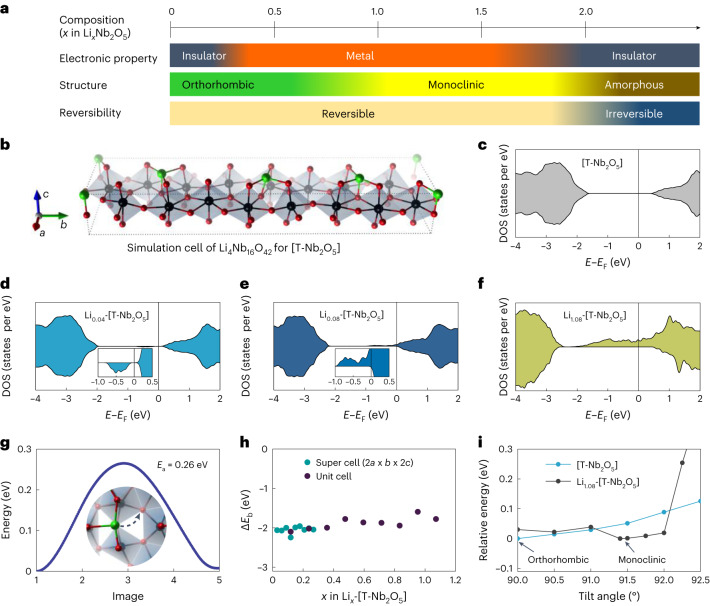
Electronic and structural phases of *T*-Nb_2_O_5_ via Li intercalation. **a**, Electronic and structural phase diagram of *T*-Nb_2_O_5_ film versus Li intercalation. The phase transitions and their reversibility are presented schematically. **b**, Structural model of the orthorhombic model system (Li_4_Nb_16_O_42_), which was employed to simulate the conventional (pristine) unit cell of *T-*Nb_2_O_5_ (Nb_16.8_O_42_, shown in Supplementary Fig. 16), in which 4 Li are added per unit cell to replace the charge of the fractionally distributed 0.8 Nb atom/cell within the 4*g* layer. This model for *T-*Nb_2_O_5_ characterized by a Li_4_Nb_16_O_42_ stoichiometry is considered as unlithiated and referred to as [*T-*Nb_2_O_5_] throughout. **c**, Total density of states (DOS) of the [*T-*Nb_2_O_5_] model unit cell. **d**,**e**, Total DOS with one and two extra Li intercalated into the (*a* × *b* × 3*c*) supercell of our simulation model (that is, Li_0.04_-[*T-*Nb_2_O_5_] (**d**) and Li_0.08_-[*T-*Nb_2_O_5_]) (**e**). **f**, Total DOS of the monoclinic Li_1.08_-[*T-*Nb_2_O_5_] structure. **g**, Diffusion performance of the one extra Li in the primitive Li_0.12_-[*T-*Nb_2_O_5_] unit cell, including free-energy profile and diffusion barrier (*E*_a_). **h**, Evolution of the differential binding energy (Δ*E*_b_) for low Li concentration within the supercell (2*a* × *b* × 2*c*) and at high concentration of Li within the primitive [*T-*Nb_2_O_5_] unit cell. **i**, The relative energy evolution of two lithiation states ([*T-*Nb_2_O_5_] and Li_1.08_-[*T-*Nb_2_O_5_]) as a function of the monoclinic angle in the range between 90° and 92.5°.

**Fig. 4:**
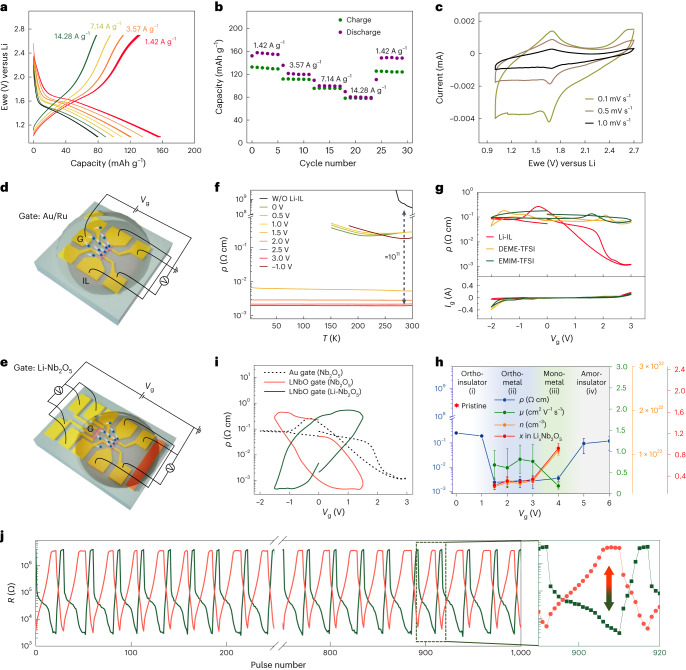
Electrochemical and electronic properties of epitaxial *T*-Nb_2_O_5_ thin films. **a**,**b**, Galvanostatic discharge–charge curves (**a**) and specific capacity obtained at each cycle (**b**) at different current rates for a 40 nm *T*-Nb_2_O_5_/LSAT(110). An irreversible capacity is observed at the slowest current densities, which is attributed to corrosion of parts of the device. **c**, Cyclic voltammogram recorded at different scan rates. The second cycle of each cycling rate is shown. **d**,**e**, Schematics of the ILG device with the Au gate electrode (**d**) and using the LNbO gate electrode (**e**). G represents gate electrode. Blue spheres denote Li-ion migrations from gating. **f**, Temperature-dependent resistivity curves at different gate voltages. **g**, *V*_g_-dependent resistivity and leakage current curves for several ILs. The sweeping rate was 16 mV s^−1^. **h**, *V*_g_-dependent carrier concentration (*n*), Li composition (*x*), mobility (*μ*) and resistivity (*ρ*) curves from Hall measurements at 200 K for Li_*x*_Nb_2_O_5_. The mean and standard deviation are represented with the error bars after three measurements. The resistivity of the pristine film is indicated by the red pentacle. Multistep transitions are shown with increasing *V*_g_, that is, (i) orthorhombic insulator (white regime), (ii) orthorhombic metal (blue), (iii) monoclinic metal (red) and (iv) insulating state (grey). The carrier concentration suddenly increases, and the mobility starts to decrease at ~4 V, indicating carrier scattering at high Li concentrations. The Li concentrations are obtained from Hall measurements, assuming that each Li atom creates one charge carrier. **i**, *V*_g_-dependent *ρ* curves for the 16 nm *T*-Nb_2_O_5_/LSAT(110) devices using the LNbO gate electrode (blue and light blue curves) and the Au electrode (the dotted black curve). The sweeping rate was 16 mV s^−1^. **j**, Pulse voltage gating of twin *T*-Nb_2_O_5_ devices. The device structure is depicted in **e**. Pulse voltages of 3 V/−3 V were applied with a pulse with of 50 ms. The channel resistances were measured at 1 µA (*I*_sd_). The film thickness and channel size were 30 nm and 60 × 30 µm^2^, respectively.

The correlation between the in situ powder X-ray diffraction/SXRD and in situ thin-film X-ray diffraction measurements allows us to study the structural evolution as a function of Li concentration and *V*_g_ (Fig. 2f). The single-crystalline thin-film morphology enables monitoring of the change in electrical resistance as a function of Li-ion concentration and carrier mobility via Hall effect measurements (described later in Fig. 4h). These measurements therefore allow the correlation of the film’s electronic properties with its structure. Finally, the Li-ion concentration-dependent electronic and structural phase diagram of the *T-*Nb_2_O_5_ thin film is obtained (Fig. 3a), revealing multistep phase transitions between the initial orthorhombic insulator (*x* < 0.3), an orthorhombic metal (0.3 ≤ *x* < 0.8), a monoclinic metal (0.8 ≤ *x* < 1.8) and an irreversible transition to an amorphous insulator (*x* ≥ 1.8). The tetragonal phase observed by in situ SXRD of the powder sample was not observed in thin films, possibly because of a clamping effect via the substrate, which hinders this phase change and thus drives the amorphization. In particular, the early lithiation stage in *T-*Nb_2_O_5_ is promising for fast and reversible electronic applications due to the sharp conductivity change without the substantial structural change.

## DFT calculations on *T*-Nb_2_O_5_ via lithiation

We investigated the evolution of the structural and electronic properties of *T-*Nb_2_O_5_ during lithiation through ab initio study. Here, we designed a new structural simulation model (Fig. 3b) for pristine *T-*Nb_2_O_5_ (Nb_16.8_O_42_) by eliminating the fractional occupancy of Nb and the resulting charge imbalance (Supplementary Section 3.2), resulting in a DFT-derived band gap of ~2.3 eV (Fig. 3c). Next, we studied the energetics and kinetics of Li interstitials in our model unit cell of [*T-*Nb_2_O_5_]. We found that Li interstitials are located within the 4*g* layer atop two-coordinated oxygen atoms with a marginally stronger binding energy (Δ*E*_b_ = −2.06 eV, defined in Methods) than that of other sites (Supplementary Section 3.3). Then, we investigated Li diffusion along the minimum energy path from this site to sites atop neighbouring O atoms (Fig. 3g) and found an activation barrier of *E*_a_ = 0.26 eV. Since the diffusion barriers and energy differences for Li at different sites are both small, there is a high probability that the incorporated Li ions are not trapped in fixed locations.

To avoid spurious interactions between periodic images along the *c* direction, which has a small lattice periodicity, we model low Li concentrations by using supercells. With one extra Li inserted into an (*a* × *b* × 3*c*) supercell, the band gap is found to be greatly reduced (Fig. 3d) from 2.3 eV (in [*T-*Nb_2_O_5_]) to 0.3 eV (in Li_*x*=0.02_-[*T-*Nb_2_O_5_] (*x* = Li/Nb)), because a new, filled defect band just below the Fermi energy (*E*_F_) is induced by this Li interstitial (Supplementary Fig. 17b). After that, the material becomes metallic once a second extra Li is intercalated into the (*a* × *b* × 3*c*) supercell, as shown in Fig. 3e. This is because the newly induced band crosses the *E*_F_ (Supplementary Fig. 17c). In addition, we note that the size of the simulation supercell (especially that of the *c* axis) affects the Li concentration required to induce metallization (Supplementary Section 3.4). Therefore, ab initio calculations can provide a qualitative explanation for the rapid onset of metallization at low Li concentration.

Finally, we investigated the differential binding energy (Δ*E*_b_) as a function of Li interstitial concentration in our [*T-*Nb_2_O_5_] model. For low Li concentrations (*x* = Li/Nb < 0.2), the obtained Δ*E*_b_ fluctuates around −2 eV (−1.95 eV to −2.24 eV) (Fig. 3h). With increasing Li concentration, the magnitude of Δ*E*_b_ gradually decreases, but it remains smaller than −1.5 eV until at least Li/Nb = 0.5. Moreover, we found that at higher Li concentration (Li/Nb ≈ 0.5) a phase transformation from orthorhombic to monoclinic is energetically favourable. Figure 3i shows the relative change of the total energy of the Li_*x*=0.54_-[*T-*Nb_2_O_5_] structure as a function of the monoclinic tilt angle varying between 90° and 92.5°. The monoclinic phase with a tilt angle of about 91.4° is more stable than the orthorhombic one by 0.44 meV per atom, and it is consistent for other metastable configurations of Li_*x*=0.54_-[*T-*Nb_2_O_5_] (Supplementary Figs. 20 and 21). Entropy corrections at the finite temperature (300 K) originating from phonon contributions also stabilize the monoclinic phase by additional 0.58 meV per atom (for a total free-energy difference of 1.02 meV per atom) compared with the orthorhombic phase. For comparison, we also considered the stability of the monoclinic phase for the case of the pristine unlithiated [*T-*Nb_2_O_5_] unit cell (Fig. 3i) and found that in this case the orthorhombic phase is more stable. Therefore, the calculations predict that *T-*Nb_2_O_5_ will undergo a phase transition from orthorhombic to monoclinic at Li/Nb ≈ 0.5, in agreement with our experimental findings.

## Electrochemical and electronic properties via Li intercalation

Epitaxial *T*-Nb_2_O_5_ single-crystalline thin films deposited on LSAT(110) substrates were characterized electrochemically in a typical Li-ion battery configuration allowing the quantification of inserted Li depending on the reaction speed. Li metal and carbonate-based electrolyte with LiPF_6_ salt were used as anode/reference electrode and electrolyte, respectively (Methods and Supplementary Fig. 22). Galvanostatic cycling at current densities ranging from 1.43 to 14.3 A g^−1^ delivered the expected voltage versus capacity profile, with reversible capacities ranging from 130–80 mAh g^−1^ (Fig. 4a,b). This indicates another figure of merit representing the excellent intercalation kinetics for the epitaxial film in accord with previous reports on nanoparticles^34^. The electrochemical properties were also demonstrated by cyclic voltammetry for 16, 80 and 160 nm thick films (Fig. 4c and Supplementary Fig. 23).

To explore the electronic property changes of epitaxial *T*-Nb_2_O_5_ thin films upon lithiation, Hall devices for Li-ILG were fabricated with the Au/Ru gate electrode (Fig. 4d and Supplementary Fig. 24a). Before placing 0.3 M Li-TFSI in EMIM-TFSI (Li-ionic liquid (IL)) onto the device, we first measured temperature-dependent resistivity (RT) curves of pristine *T*-Nb_2_O_5_ films using a high resistance meter. The room temperature resistivity of the *T*-Nb_2_O_5_ film is 2.78 × 10^8^ Ω cm (Supplementary Fig. 25), which is comparable with previous reports ranging from 10^7^ to 10^9^ Ω cm (refs. ^43,44^). After placing IL onto the device, the resistivity drops to ~5 × 10^−1^ Ω cm due to the lower resistance of the IL (~10^14^ Ω for *T*-Nb_2_O_5_ while ~10^6^ Ω for IL when a source-drain current of 1 µA is applied; Supplementary Fig. 26). Therefore, before Li intercalation, the resistance of the device is determined by the resistance of the IL. We emphasize that the high limit of the resistivity accessible to the *T*-Nb_2_O_5_ material is given by the ex situ measured value, while at the low limit the resistivity is governed by the gated *T*-Nb_2_O_5_ film. The source-drain current- (*I*_sd_) or voltage (*V*_sd_)-dependent resistance changes of IL alone show that the resistance of the IL decreases as increasing *I*_sd_/*V*_sd_ (Supplementary Fig. 26), which is probably due to the leakage from IL decomposition at high voltages. Therefore, by adjusting the operating parameters of our device (for example, using a lower *I*_sd_/*V*_sd_), we can measure a larger resistivity change during gating. Note that, for simplicity of the resistivity calculations of the device, all resistivities were calculated using the film thickness and the channel dimensions of 65 × 30 µm^2^. The RT curves at different gate voltages are shown in Fig. 4f. Notably, the resistivity after metallization is ~2 × 10^−3^ Ω cm, which is approximately 11 orders of magnitude smaller than that of pristine *T*-Nb_2_O_5_. Such a colossal insulator–metal transition is remarkable compared to the previously reported metallization by ILG^1–13^ (Supplementary Fig. 28 and Supplementary Table 3).

To investigate the origin of metallization, the devices were gated between *V*_g_ = 3 and −2 V using conventional ILs, including DEME-TFSI, EMIM-TFSI and 0.3 M Li-TFSI in EMIM-TFSI (Li-IL) (Fig. 2g). While Li-ILG induces metallization, no noticeable change in resistivity was observed for pure IL. This contrasts with ILG of other oxide films, such as VO_2_, WO_3_ and SrCoO_2.5_, all of which show metallization in the absence of Li ions due to oxygen or hydrogen ion migration^2–10^. The effect of the substrate orientation and film thickness on ILG is summarized in Supplementary Fig. 29. All films grown on (001)- and (110)-oriented substrates show good metallization behaviour owing to vertical ionic transport channels. For thicknesses ranging from 16 to 160 nm and gating with sufficiently slow voltage sweep rates, the resistivity at *V*_g_ = 3 V is close to 10^−3^ Ω cm, demonstrating that the metallization happens in the bulk of the material. This is further confirmed by hard X-ray absorption near-edge structure (XANES) measurements (Supplementary Fig. 30), where Nb K- absorption edge shifts to lower energy upon gating to positive potentials. This indicates the reduction of the Nb oxidation state in the bulk of the film, in agreement with prior work on a polycrystalline sample^45^. When we performed high voltage gating (Supplementary Figs. 31 and 32), the resistivity increased as the gating voltage was increased above 4.5 V, indicating the onset of a metal-to-insulator transition. The voltage-dependent carrier concentration, mobility and resistivity changes are summarized in Fig. 4h. The electronic transitions correlate well with the in situ X-ray diffraction data in Fig. 2a,f.

## Tunable and low voltage operation by chemical potential control

The working voltage of a Li-ion battery cell depends on the difference in chemical potential between the cathode and the anode^18^. To exploit this, the Au gate electrode was replaced by conventional Li-containing electrode materials, namely LiFePO_4_ (LFP), LiCoO_2_ (LCO) and Li_*x*_Nb_2_O_5_ (LNbO). The Li intercalation potentials in these materials are ~3.5, 4.0 and 1.8 V versus Li^+^/Li for LFP, LCO and LNbO, respectively, and are all within the electrochemical stability range of the IL used in this study. The fabrication process is described in Supplementary Section 6 and Fig. 4e, and the *V*_g_-dependent resistivity (RV) curves with different gate electrodes are shown in Supplementary Fig. 35. The critical metallization voltage (*V*_c_) is defined from the peak of the normalized d*ρ*/d*V* curves. The chemical potential (V versus Li/Li^+^)-dependent *V*_c_ plot (Supplementary Fig. 35c) shows that *V*_c_ tends to decrease as the potential difference between the gate electrode and *T*-Nb_2_O_5_ decreases. Notably, the LNbO gate electrode leads to coupled resistivity changes between the LNbO gate electrode and Nb_2_O_5_ channel due to the ion exchange between them, and it reveals a substantial decrease in the *V*_c_ value of ~0.54 V compared to the Au electrode (Fig. 4i), which has been extensively used in typical ILG devices^2–5^. In particular, such potential control of *V*_c_ is not possible with conventional ILG based on proton insertion or oxygen ion loss, because the *V*_c_ will be determined by the more poorly determined electrochemical process that occurs at the counter (gate) electrode (including double layer formation, electrolyte degradation and oxygen evolution).

## Pulsed voltage gating of *T*-Nb_2_O_5_ and WO_3_ thin-film devices

We performed pulsed voltage gating on *T*-Nb_2_O_5_ and WO_3_ thin-film devices to compare the kinetics of Li insertion and metallization (Supplementary Section 7). To explore the effects of the crystal orientation and the presence of crystallinity, devices were fabricated using an Au gate electrode, as depicted in Fig. 4d, for a 30 nm polycrystalline *T*-Nb_2_O_5_ thin film grown on a YSZ(001) substrate (Supplementary Fig. 36) and compared to the single-crystalline thin films deposited on LSAT(110) having the same thickness (Supplementary Fig. 39). The single-crystalline *T*-Nb_2_O_5_ device shows metallization after a single pulse of 4.3 V with a pulse width (*W*) of 0.8 s (Supplementary Fig. 39) while the polycrystalline *T*-Nb_2_O_5_ becomes metallic after 45 pulses, indicating that the vertical orientation of ionic channels plays a crucial role for the fast ionic gating. The critical resistivity for metallization was defined as 2 × 10^−3^ Ω cm, which shows a decrease in resistivity with decreasing temperature (Fig. 4f and Supplementary Fig. 38b). The pulse voltage (*H*)-dependent resistance changes of the single-crystalline *T*-Nb_2_O_5_ device are shown in Supplementary Fig. 37. Moreover, a 30 nm epitaxial WO_3_ film (Supplementary Fig. 38) was gated with Li-IL at 3.5 V and pure IL (DEME-TFSI) at 4.3 V and shows metallization after 8 and ~1,500 pulses, respectively, showing that Li^+^ ion gating is much faster than the O^2−^/H^+^ ion gating (Supplementary Fig. 39). Note that pulsed voltage gating above 4 V on WO_3_ with Li-IL leads to increased resistance compared to 3.5 V (Supplementary Fig. 38c), which is probably due to a conversion reaction^31^, limiting higher voltage operation. Single-crystalline *T*-Nb_2_O_5_ features a resistance change by ~11 orders from the initially insulating film to the gated metallic film by a single pulse, while for WO_3_ the change is ~6 orders of magnitude (Supplementary Fig. 39b). The pulsed width-dependent resistance changes (Supplementary Fig. 40) further reveal the larger resistance changes of the single-crystalline *T*-Nb_2_O_5_ thin film compared to the WO_3_ thin film in the electrochemical reaction region.

The single-crystalline *T*-Nb_2_O_5_ device with an Au gate electrode reveals one order of magnitude resistance change over ~3.5 × 10^5^ pulses (3.8 V/−2 V with a pulse width of 50 ms), illustrating good reversibility (Supplementary Fig. 41). In particular, by replacing the Au gate electrode with Li-Nb_2_O_5_ (Fig. 4e), coupled responses between twin *T*-Nb_2_O_5_ devices are realized (Fig. 4j). The resistances of the two devices oscillate out of phase for three orders of magnitude for more than 10^3^ pulses, when applying 3 V/−3 V and a pulse width of 50 ms.

In summary, we have realized the growth of single-crystalline epitaxial *T*-Nb_2_O_5_ thin films with vertically oriented 2D channels that provide paths for fast ionic migration. This morphology allows us to study the evolution of the electronic and structural properties during Li-ILG by comprehensive in situ and ex situ experiments. They reveal unexplored sequential phase transformations, including an orthorhombic insulator, an orthorhombic metal, a monoclinic metal and a degraded insulating phase, as Li concentration is increased. DFT calculations further support that the monoclinic phase (approximately Li_1_Nb_2_O_5_) is energetically favourable and metallic. Defect electronic states near the Fermi energy from Li-ion migration lead to abrupt changes in resistivity. Thus, the *T*-Nb_2_O_5_ films with vertical ionic transport channels undergo substantial electrical change in an early stage of Li insertion into the initially insulating *d*^0^ films, leading to a colossal insulator–metal transition with a change in resistance of 11 orders of magnitude. The *T*-Nb_2_O_5_ film shows an even larger and faster resistance change and a wider voltage operation range via Li interaction, compared with WO_3_ thin films, which are one of the best electrochemical materials. Moreover, coupled electronic responses between twin *T*-Nb_2_O_5_ devices are demonstrated through ionic exchange between each other. This work showcases a synergistic experiment–theory approach to develop new ionically channelled devices for diverse applications, including thin-film batteries, electrochromic devices, neuromorphic devices and electrochemical random-access memory.

## Methods

### Film growth

A RHEED-assisted PLD system using a 248 nm KrF excimer laser was employed to optimize the growth conditions of Nb_2_O_5_ thin films by varying the growth temperature from 500 to 900 °C. The laser fluence, oxygen partial pressure (*p*O_2_) and repetition rate were 1 J cm^−2^, 10 mT and 6 Hz, respectively. The optimized growth temperature of *T*-Nb_2_O_5_ thin films was ~620 °C. The heating and cooling rates were 30 and 10 °C min^−1^, respectively. Substrates of LSAT(001), LAO(001), LSAT(110) and LAO(110) were used to study substrate orientation-dependent domain structures. A YSZ(001) substrate was used to grow a polycrystalline *T*-Nb_2_O_5_ thin film. For gate electrode potential control, LFP and LCO thin films were grown on LSAT(110) substrates by varying *p*O_2_ at room temperature. The laser fluence and repetition rate were 1 J cm^−2^ and 6 Hz, respectively. The optimal *p*O_2_ was 1 mT and 10 mT for the LFP and LCO thin films, respectively, as determined by X-ray photoelectron spectroscopy (XPS) characterization. A WO_3_ thin film was grown on a LAO(001) substrate. The growth temperature, laser fluence, oxygen partial pressure (*p*O_2_) and repetition rate were 600 °C, 1 J cm^−2^, 200 mT and 6 Hz, respectively. The film thickness was characterized by X-ray reflectivity measurements or fringes of theta–2theta scans, and the *T*-Nb_2_O_5_ thickness was further confirmed by TEM measurements.

### Ionic liquid gating device fabrication

Standard photolithographic techniques were used to fabricate ionic liquid gating devices. Substrates with a size of 5 × 5 mm^2^ were used. A *T*-Nb_2_O_5_ channel with a size of 65 × 30 μm^2^ was etched and then Ru (5 nm) and Au (70 nm) layers were successively deposited using ion beam sputtering (SCIA coat 200) for both the gate electrode and channel contacts. The IL covered both the *T*-Nb_2_O_5_ and the gate electrode. For the gate electrode potential control, Li ion-containing oxides were deposited on top of the Au/Ru gate electrode.

For the LNbO gate electrode device fabrication, both channel and gate were etched, and then Au (70 nm)/Ru (5 nm) layers were deposited to make channel and gate contacts. Then, the reference electrode (LFP) was deposited using pulsed laser deposition. After the device fabrication, Li ions were moved from the LFP to the gate electrode by ILG to make the LNbO gate electrode. Then, the gating was applied to the Nb_2_O_5_ channel using the LNbO gate electrode.

For the in situ X-ray diffraction measurements of the thin films, a *T*-Nb_2_O_5_ channel with a dimension of 2 × 2 mm^2^ was etched, then Au (70 nm)/Ru(5 nm) layers were deposited for the gate electrode and channel contacts. The device was then attached using double-sided tape to a specially designed sample holder. The IL was placed on the device surface, and then Kapton film was attached to reduce the thickness of the IL. Resistance and *θ*–2*θ* scans were measured during in situ ILG.

### Thin-film characterization

The *θ*–2*θ* scans, phi scans and in situ X-ray diffraction on the thin films were carried out using a Bruker D8 Discovery X-ray diffractometer with CuKα radiation. Reciprocal space map measurements were performed using a Ga jet X-ray source (*λ* = 1.34 Å) and a six-circle diffractometer equipped with a Pilatus 100 K pixel detector. HAADF-STEM imaging was performed using a JEOL ARM200F with a spherical aberration corrector (CEOS) operated at 200 kV. XPS (K-Alpha, Thermo Scientific) was conducted for Li ion-containing oxides. The film surface was gently cleaned by cluster ion etching before the measurement.

Diethylmethyl(2-methoxyethyl)ammonium bis(trifluoromethylsulfonyl)imide (DEME-TFSI), 1-ethyl-3-methylimidazolium bis(trifluoromethylsulfonyl)imide (EMIM-TFSI) and Li-IL were used for the ILG. For the Li-IL, a lithium bis(trifluoromethanesulfonyl)imide (Li-TFSI) powder was dissolved in the EMIM-IL at 50 °C for 2 h to achieve a solution of molality 0.3 mol kg^−1^. Each IL was dried in a vacuum chamber (10^−6^ mbar) at 105 °C for at least 10 h before use.

Transport measurements of the ILG devices were carried out in a physical property measurement system (Quantum Design). Gate voltages were applied using a Keithley 2450A source meter. For the resistance measurements, a constant current of 1 µA was applied using a Keithley 6221 current source, and the voltage was measured by a Keithley 2182A nano-voltmeter. The gate voltage was applied between the gate electrode and the *T*-Nb_2_O_5_ channel while monitoring the resistance of the channel. The substrate is insulating, thus the voltage was applied through the IL, resulting in Li-ion migration from gating. The resistance of the pristine *T*-Nb_2_O_5_ and WO_3_ thin films was measured by a high resistance meter (B2985A, KEYSIGHT). A total of 1,000 data points were averaged at each temperature in a probe station. Li-ILG of thin films for the ex situ X-ray diffraction, STEM and X-ray absorption near-edge structure measurements were carried out using a polytetrafluoroethylene boat with an Au plate (the gate electrode) covered with the Li-IL.

The pulsed voltage gating was performed in a probe station (CRX; Lake Shore). Gate voltages were applied using a Keithley 2636B source meter. A constant current of 1 µA was applied using a Keithley 6221 current source, and the voltage was measured by a Keithley 2182A nano-voltmeter to measure the channel resistance. The 6221/2182A system is advantageous for the fast readouts after the pulsed gating and provides electrical floating between each device.

The Nb K-edge X-ray absorption spectra were acquired at the CLÆSS beamline at the ALBA synchrotron^46^. The synchrotron radiation emitted by a wiggler source was monochromatized using a double crystal Si(311) monochromator, while higher harmonics were rejected by proper choice of angles and coatings of the collimating and focusing mirrors. The samples were mounted in a liquid nitrogen cryostat, and the spectra were recorded in fluorescence mode at 80 K by means of a multichannel silicon drift detector. The sample normal and the fluorescence detector were at 60 and 90 degrees with respect to the incoming beam, respectively. The fluorescence detector dead time was kept around 4.5% at 19,600 eV for both samples for a better comparison.

### Powder X-ray diffraction

Free-standing electrodes for in situ X-ray diffraction measurements with coupled electrochemistry were prepared by mixing 90 wt% *T*-Nb_2_O_5_ powder (Sigma-Aldrich), 5 wt.% polytetrafluoroethylene binder, 5 wt% carbon black (Timcal SuperP). The mixed powder was pressed and rolled onto a flat surface to give a homogeneous film. The film was formed into a disc with a diameter of 13 mm and dried in a Büchi oven at 100 °C under dynamic vacuum (10^–2^ mbar) for 12 h before transferring into an argon-filled glovebox. A customized electrochemical cell equipped with a Be window was used to prepare cells for in situ X-ray diffraction. The 70 μl of LP57 electrolyte (1 M LiPF_6_, ethylene carbonate/ethyl methyl carbonate (3/7), SoulBrain) was added to the film followed by 16 mm glass fibre separator. Battery cycling was conducted using a Land cycler at room temperature between open circuit voltage and 0.005 V at C/34 rate (34 hours for a full charge–discharge). In situ X-ray diffraction data were collected at 300 K on a Panalytical Empyrean diffractometer equipped with a Ni filter using CuKα radiation (*λ* = 1.5406 Å) in Bragg–Bentano geometry. Ex situ SXRD measurement were performed in Kapton capillaries at 11-BM beamline of the Advanced Photon Source (APS). Lithiated Li_1.2_Nb_2_O_5_ and Li_1.6_Nb_2_O_5_ powders were prepared in a swagelok type cell using LP57, glass fiber and Li metal as electrolyte, separator and counter electrode, respectively. In situ SXRD was performed in half cell configuration using the Ampix operando cell at the APS.

### Electrochemical characterization

For electrochemical cycling of thin films in a pouch cell, the samples were deposited on non-conducting LSAT substrates giving highly oriented single-crystalline films. For the current collector, Au was deposited/patterned on the surface of the thin film. The Cu tab was connected on the gold pattern (Supplementary Fig. 7). The other components of cell, anode, electrolyte and separator were the same as those used for the in situ powder X-ray diffraction experiments. The C rate was defined on the basis of 201.7 mAh g^−1^, that is, one electron reduction per Nb_2_O_5_. For cyclic voltammetry experiments, three different scan rates of 0.1, 0.5 and 1.0 mV s^−1^ were used. Galvanostatic charge–discharge was also performed with current densities of 14.28, 7.14, 3.57 and 1.43 A g^−1^. The thin-film capacity was determined from the dimensions (surface × thickness) and the specific capacity of *T*-Nb_2_O_5_ (175 mAh g^−1^). The film thickness was determined by X-ray reflectivity measurements (Supplementary Fig. 2b) and further confirmed by TEM measurements. The electroactive surface was considered to be delimited by the Au pattern (0.12 mm²).

### DFT calculations

The unit cell of unlithiated [*T-*Nb_2_O_5_] (Li_4_Nb_16_O_42_ in our model) is described in Supplementary Section 3.2. We modelled the diffusion of one Li ion in the unit cell of *T-*Nb_2_O_5_ by the nudged elastic band method with climbing image (CI-NEB). The free-energy profile of the most favourable diffusion pathway has been shown in Fig. 3g.

The differential binding energy (Δ*E*_b_) of each Li interstitial reaction in the [*T-*Nb_2_O_5_] model (as shown in Fig. 3h) was calculated as:

$$\begin{array}{l}\Delta {E}_{\mathrm{b}}=E({{\rm{Li}}}_{x}{\hbox{-}}[{\mathrm{T}}{\hbox{-}}{{\rm{Nb}}}_{2}{{\rm{O}}}_{5}])-E({{\rm{Li}}}_{x{\prime} }{\hbox{-}}[{\mathrm{T}}{\hbox{-}}{{\rm{Nb}}}_{2}{{\rm{O}}}_{5}])-0.5(x-x{\prime} )\\ \qquad \quad \times \, {n}_{{\rm{Nb}}}E({\rm{Li}})\,({n}_{\mathrm{Nb}}=16.8\,{\rm{or}}\,67.2)\end{array}$$

where *n*_Nb_ indicates the number of Nb atoms in the cell, and 0.5(*x* − *x*′) × *n*_Nb_ is the number of extra Li atoms intercalated into the material. *E* indicates the DFT energies of the compositions calculated from ab initio studies. The reference energy of *E*(Li) is calculated from bulk Li metal. The energy of [*T-*Nb_2_O_5_] is calculated from our unit cell model of Li_4_Nb_16_O_42_ and supercell of Li_16_Nb_64_O_168_, respectively. All possible interstitial sites for each Li atom were considered and the site providing the most negative Δ*E*_b_ is the most energetically favourable location for that atom (these sites are shown in Supplementary Fig. 20). Such an arrangement of interstitial Li was used iteratively as a starting configuration for the higher concentration simulations. More detailed computational methods are shown in Supplementary Section 3.1.

### Reporting summary

Further information on research design is available in the Nature Portfolio Reporting Summary linked to this article.

## Online content

Any methods, additional references, Nature Portfolio reporting summaries, source data, extended data, supplementary information, acknowledgements, peer review information; details of author contributions and competing interests; and statements of data and code availability are available at 10.1038/s41563-023-01612-2.

## Supplementary information

Supplementary Information Supplementary Sections 1–8, Figs. 1–41 and Tables 1–3.

Reporting Summary

Supplementary Data 1 Structural data for calculations.

## Data Availability

The main data that support the results of this study are available within this Article and its Supplementary Information.
