# Supplementary material for: Li iontronics in single-crystalline T-Nb2O5 thin films with vertical ionic transport channels
Source: Nat Mater. 2023 Jul 27;22(9):1128–35. doi: 10.1038/s41563-023-01612-2 (PMC10465368; doi:10.1038/s41563-023-01612-2)
Supplement: Supplementary file 1 — Supplementary Sections 1–8, Figs. 1–41 and Tables 1–3. [file 41563_2023_1612_MOESM1_ESM.pdf]

nature materials

Article

https://doi.org/10.1038/s41563-023-01612-2

Li iontronics in single-crystalline T-Nb_2O_5 thin films with vertical ionic transport channels

In the format provided by the authors and unedited

Table of Contents

1. Structural analysis of pristine *T*-Nb_2O_5 thin films

2. *In situ* and *ex situ* structural analysis via lithiation

3. Computational results

4. Electrochemical tests of *T*-Nb_2O_5

5. Transport properties of *T*-Nb_2O_5 thin films via lithiation

6. Gate voltage tuning by using Li-containing gate electrodes

7. Electronic device performance

8. References in Supplementary Information

1. Structural analysis of pristine *T*-Nb_2O_5 thin films

Niobium pentoxide (Nb_2O_5) exhibits many polymorphs such as *TT*-, *T*-, *M*-, *H*-, *B*-, *N*-, *R*-Nb_2O_5[1]. Among them, *T*-Nb_2O_5 (*T*: Tief in German) is one of the fastest Li^+-ion conductors found in oxides, featuring two-dimensional 4g atomic layers which provide exceptionally fast Li ion transport paths with very low steric hindrance (Supplementary Fig. 1). The single *T*-Nb_2O_5 (180) thin film is obtained at the growth temperatures between 600 and 650 ˚C by using PLD (Supplementary Fig. 2). Phi scans of the films grown on (001)-oriented substrates show multi-domains with 4-fold symmetry that the domains are in-plane rotated 90° each other (Supplementary Fig. 4b), whereas those of films grown on (110)-oriented substrates reveal 2-fold symmetry (Supplementary Fig. 4d). The substrate-orientation dependent domain structures are consistent with the reflection high-energy electron diffraction (RHEED) patterns (Supplementary Fig. 2b and 2c) and STEM-HADDF images (Supplementary Fig. 3). Namely, the film grown on LSAT (110) shows anisotropic in-plane diffraction patterns of *T*-Nb_2O_5 $[8\bar{1}0]$ and $[001]$ which are rotated 90° each other, while the film grown on LAO (001) exhibits the mixed diffraction patterns of *T*-Nb_2O_5 $[8\bar{1}0]$ and $[001]$.

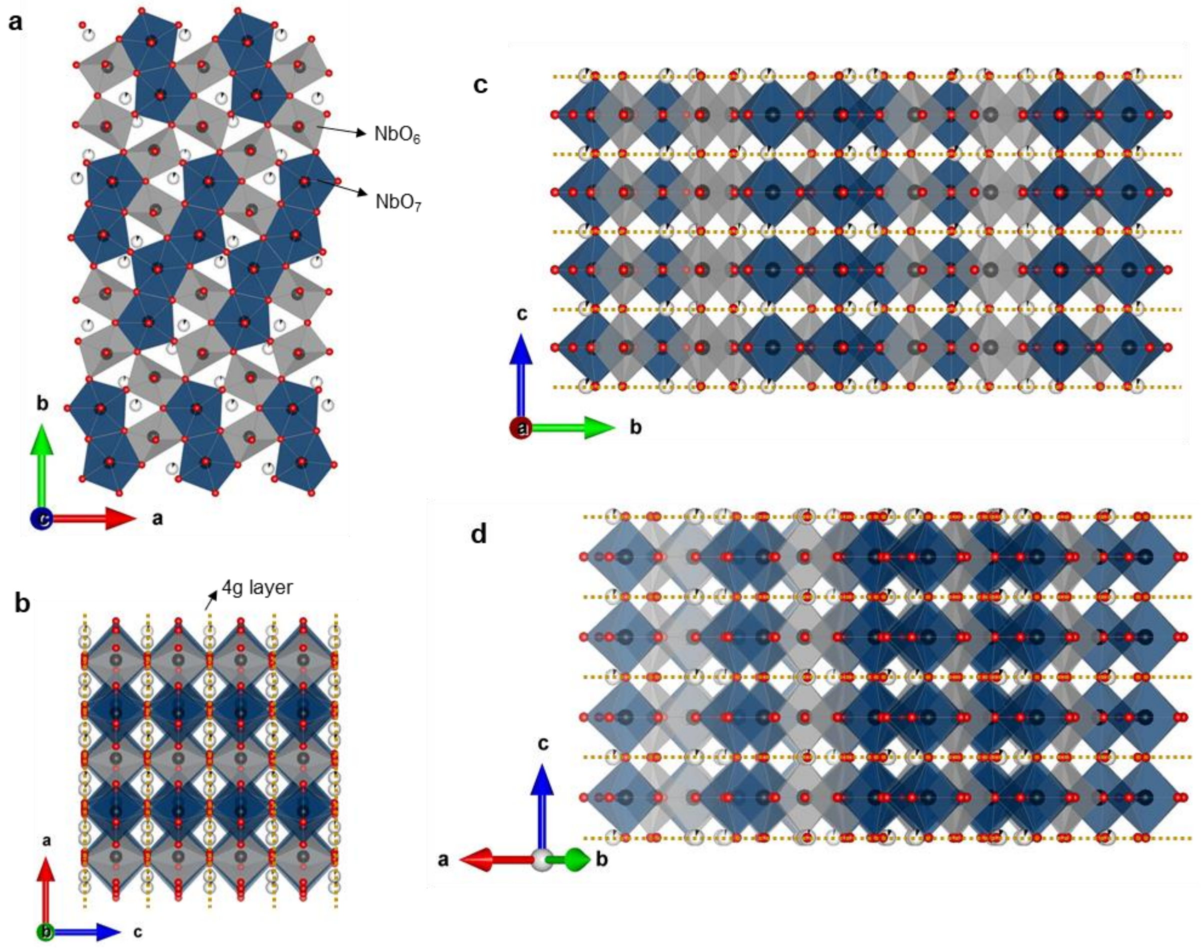

Supplementary Fig. 1. Crystal structure of *T*-Nb_2O_5. Schematic structures viewed along **a**, [001], **b**, [010], **c**, [100], and **d**, [180]. The (partially filled) navy and red spheres represent (partially occupied) Nb and O atoms, respectively. The grey and navy polyhedra denote distorted octahedra (NbO_6) and pentagonal bipyramids (NbO_7), respectively. The dashed dark yellow lines represent loosely packed 4g atomic layers.

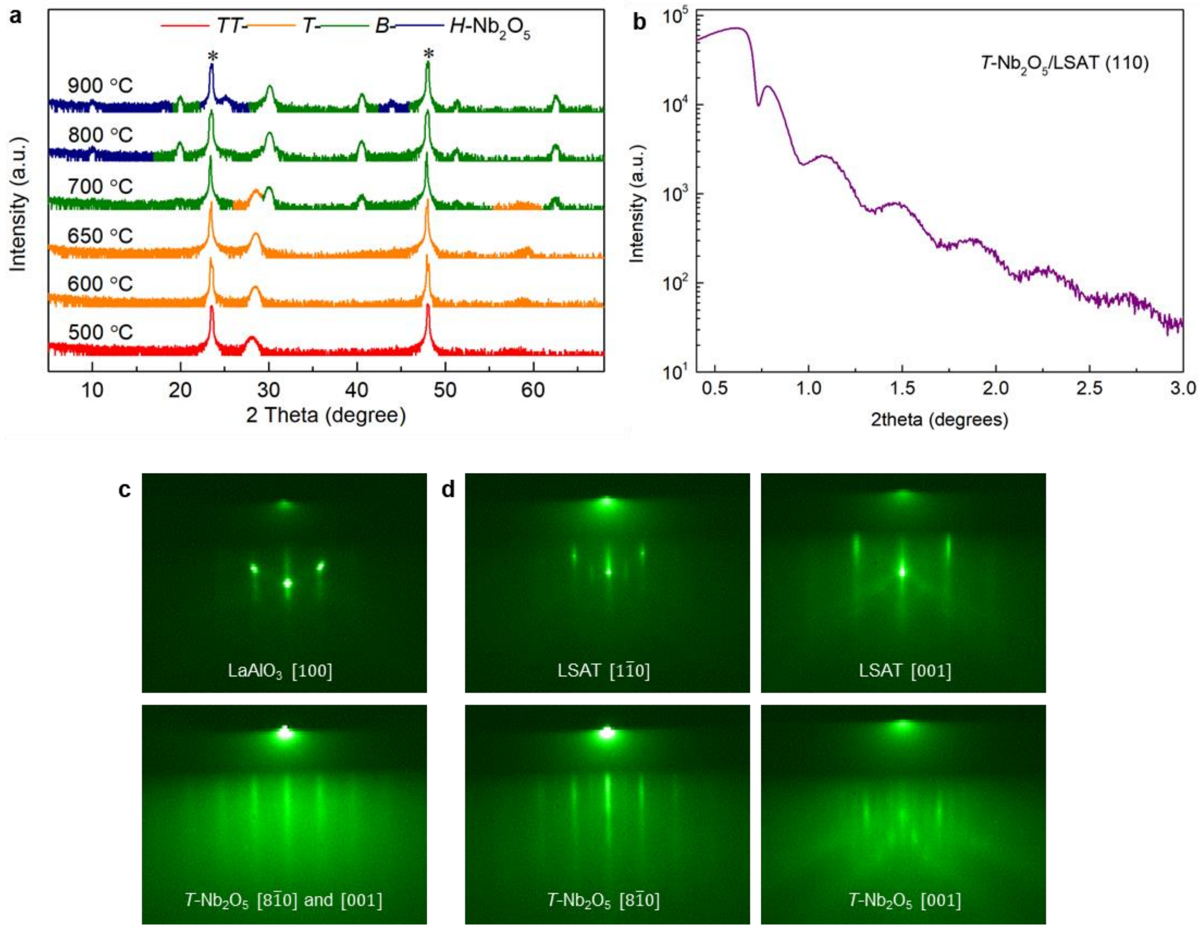

Supplementary Fig. 2. Optimization of epitaxial *T*-Nb_2O_5 thin film growth and RHEED patterns. a, Theta-2theta XRD scans with various growth temperatures grown on LAO (001) substrates. The results reveals many Nb_2O_5 polymorph formations such as *TT*-, *T*-, *B*-, and *H*-Nb_2O_5. Nonetheless, the growth temperatures between 600 and 650 °C reveal single *T*-Nb_2O_5 phase formation. **b**, X-ray reflectivity (XRR) of a *T*-Nb_2O_5 thin film grown on a LSAT (110) substrate at 620 °C. The film thickness of ≈16 nm was obtained from the periodicity of intensity oscillations. RHEED patterns of *T*-Nb_2O_5 thin films grown on **c**, LAO (001) and **d**, LSAT (110) substrates at the growth temperature of 650 °C. The top and bottom images represent before and after deposition, respectively. The film grown on the LSAT (110) substrate shows anisotropic in-plane diffractions of *T*-Nb_2O_5 [8$\bar{1}$0] and [001]. While, the film grown on the LAO (001) substrate exhibits a mixture of *T*-Nb_2O_5 [8$\bar{1}$0] and [001] diffractions, indicating existence of multi-domains with 90° rotation each other.

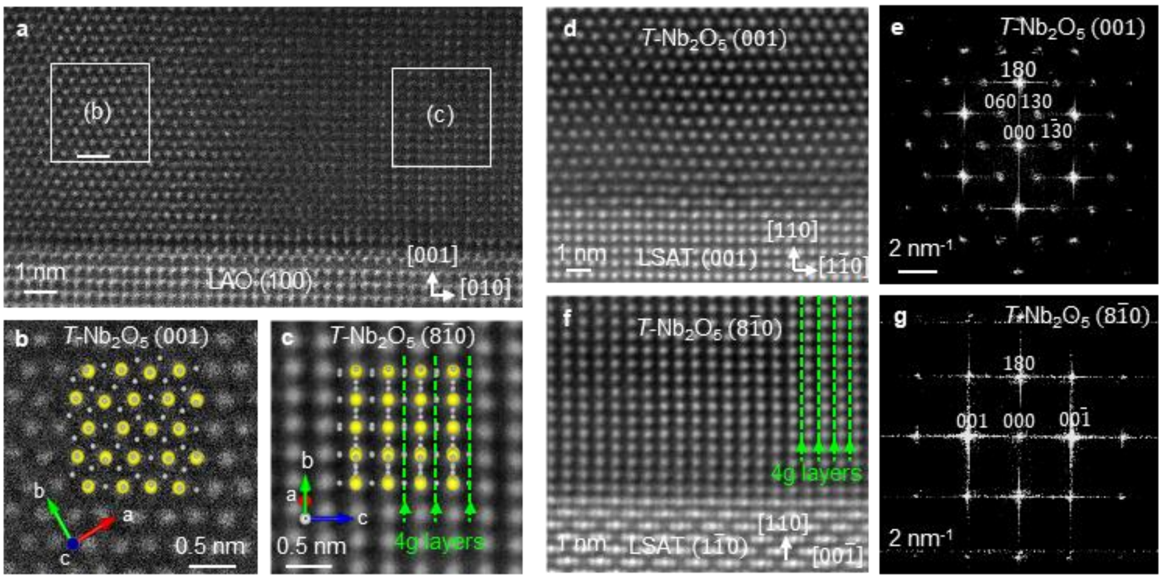

Supplementary Fig. 3. Cross-sectional HAADF-STEM images of *T*-Nb_2O_5 thin films grown on LAO (001) and LSAT (110) substrates. a, Cross-sectional HAADF-STEM image of a *T*-Nb_2O_5 thin film grown on a LAO (001) substrate viewed along LAO [100], revealing the presence of multi-domains. **b-c,** Magnified STEM images of each domain labelled in (**a**), showing domains of *T*-Nb_2O_5 [001] and [8$\bar{1}$0], respectively. Overlaid yellow and grey spheres represent Nb and O atoms, respectively. Green dashed lines represent the vertical ionic transport channels (4g layers) viewed from *T*-Nb_2O_5 [8$\bar{1}$0]. **d**, a STEM image and **e**, FFT pattern of *T*-Nb_2O_5 thin film grown on a LSAT (110) substrate viewed along the LSAT [001]. **f**, a STEM image and **g**, FFT pattern of *T*-Nb_2O_5 thin film grown on a LSAT (110) substrate viewed along the LSAT [1$\bar{1}$0].

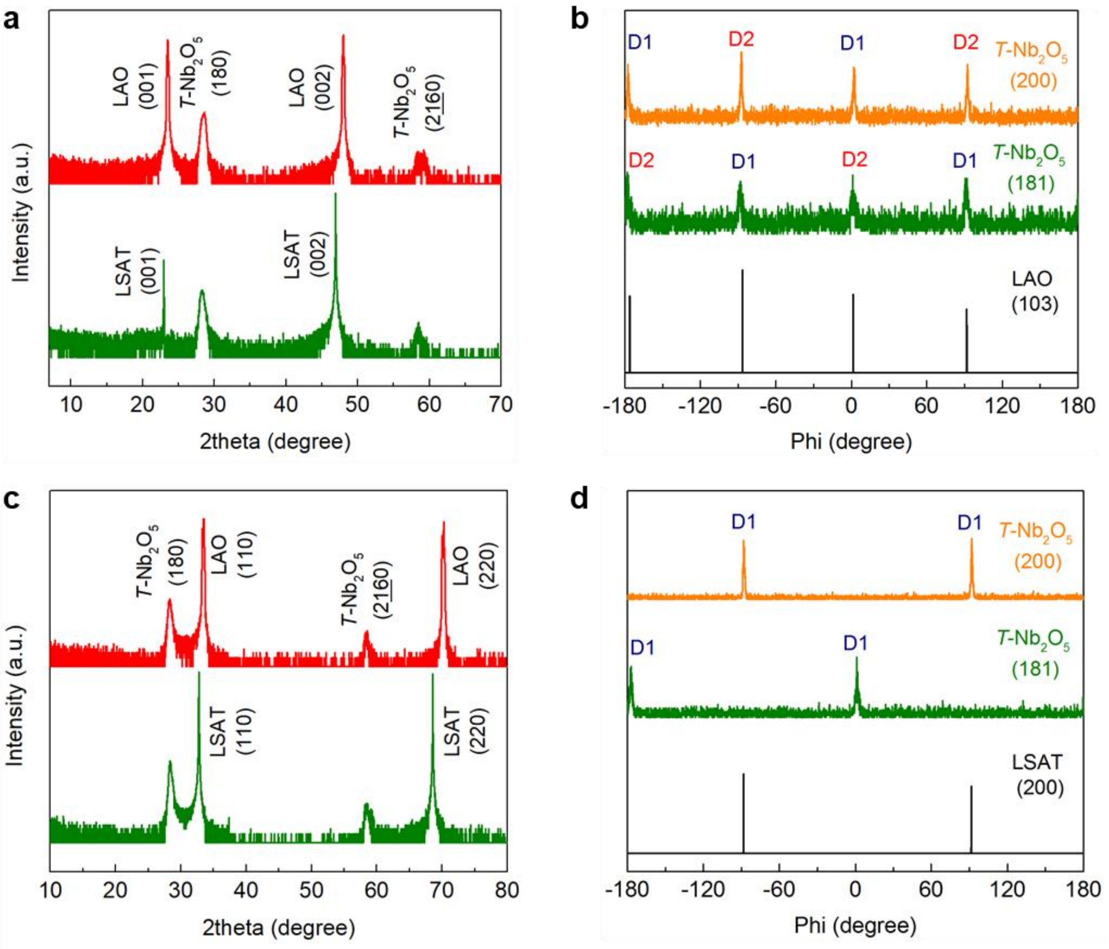

Supplementary Fig. 4. XRD characterizations of *T*-Nb_2O_5 thin films and the schematic film structures. **a**, Theta-2theta XRD scans of *T*-Nb_2O_5 thin films grown on (001)-oriented substrates such as LAO (001) and LSAT (001) substrates. **b**, Phi-scan of thin films grown on a LAO (001) substrate. The 4-fold symmetry of *T*-Nb_2O_5 (200) and (181) reflections represent the presence of multi-domains rotated 90° to each other. **c**, Theta-2theta XRD scans of *T*-Nb_2O_5 thin films grown on (110)-oriented substrates, including LAO (110) and LSAT (110) substrates. **d**, Phi-scan of thin films grown on a LSAT (110) substrate. Theta-2theta scans exhibit that all films are oriented along out-of-plane (180), and the 2-fold symmetry from phi scans of both (200) and (181) reflections are revealed.

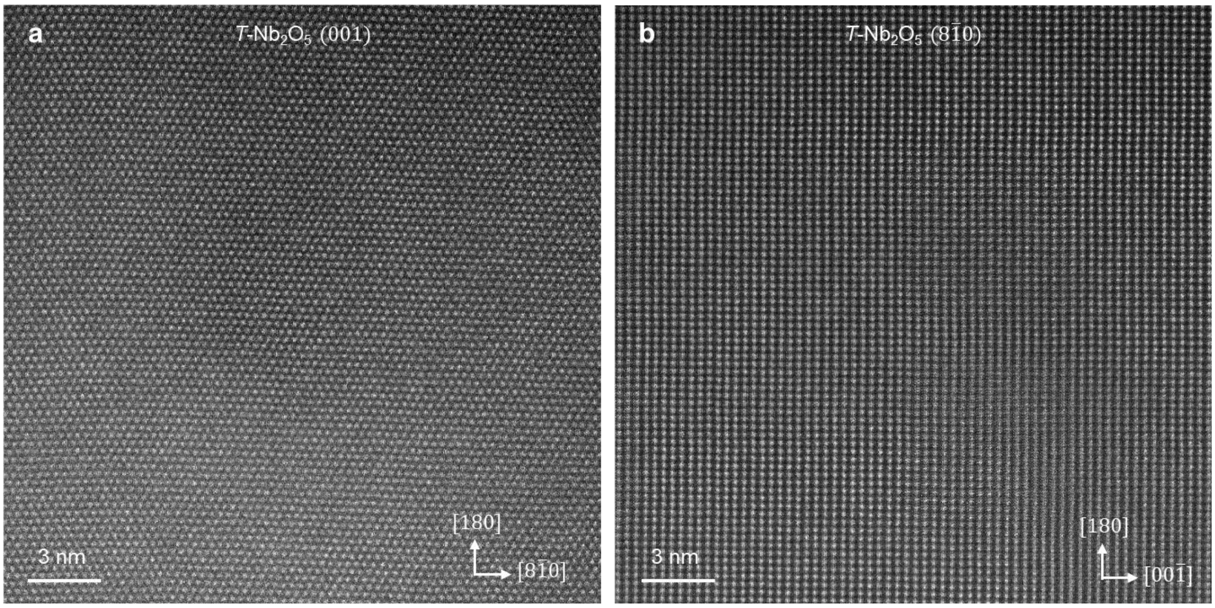

Supplementary Fig. 5. Low magnification HAADF-STEM images of single-crystalline *T*-Nb_2O_5 thin films grown on a LSAT (110) substrate. Cross-sectional STEM images of *T*-Nb_2O_5 thin film viewed along the **a**, *T*-Nb_2O_5 [001] and **b**, [8$\bar{1}$0] direction, respectively. The images reveal the single-crystalline structure.

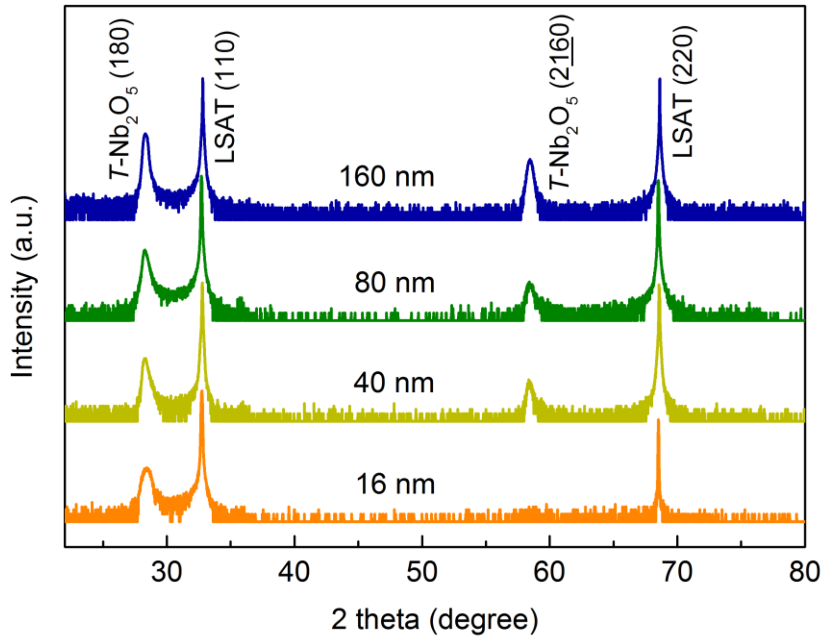

Supplementary Fig. 6. Thickness-dependent theta-2theta XRD scans of *T*-Nb_2O_5 thin films grown on LSAT (110) substrates. The XRD results show good crystallinity even at the high thickness of 160 nm.

2. *In situ* and *ex situ* structural analysis via lithiation

2.1. Monoclinic distortion of *T*-Nb_2O_5 thin films via lithiation

In Supplementary Fig. 8, *ex situ* XRD on the pristine and gated *T*-Nb_2O_5 thin films were carried out using a Gallium-metal jet x-ray source (λ=1.34 Å) and a six-circle diffractometer equipped with a Pilatus 100 K pixel detector. Reciprocal space maps (RSM) were collected under grazing incidence of the incoming beam using one set of lattice parameters for the pristine and gated sample in order to provide a relative measurement of the gating-induced modifications of the lattice of the film. We used the orthorhombic lattice characterized by a=6.12 Å, b=29.75 Å, and c= 3.89 Å and α=β=γ=90°. This lattice was used throughout the analysis, i.e. before and after gating. Therefore, we are studying relative modifications of the lattice, most importantly the change of the angle γ between the a- and the c-axis. Note that, in our setting of the film lattice this corresponds to the monoclinic angle β used in the analysis of the bulk crystal.

The effect of gating can be directly seen positions, which indicates peak shifts in the range of up to several tens of reciprocal lattice units. The analysis was carried out by calculating the length of the scattering vectors and the angles between them. RSMs were collected in the vicinity of the (200) and the (0$\underline{16}$0) reflections for the pristine (p) and the gated (g) sample. Reflection indices are given with respect to the same lattice parameters. The inset in (a) sketches a schematic emphasizing the changes of the positions of the scattering vectors. For the (200) and the (0$\underline{16}$0) reflection we find rotations of the scattering vectors by +0.7 and -2.6 degrees which add up to 3.3 degrees by which the angle γ* between the reciprocal a* and b* axis decreases. In turn this corresponds to an increase of γ by +3.3 degrees, i.e. the monoclinic angle is equal to 93.3 degrees, which is in reasonable agreement with the result derived for the bulk sample by powder x-ray diffraction.

In total we have analyzed several pairs of reflections where we find in general rotation of the scattering vector positions by up to 4 degrees, while simultaneously the magnitudes of the lattice vectors decrease between about 0.5 % and 3 % upon gating, which is related to the general lattice expansion.

2.2. Determination of the monoclinic structure for powder *T*-Nb_2O_5

In Supplementary Fig. 12 and Table 1, $Li_{1.2}Nb_2O_5$ and $Li_{1.6}Nb_2O_5$ (see methods for sample preparation) SXRD patterns could be indexed using two phases: a *Pbam* model similar to *T*-Nb_2O_5 together with a *P2/m* structural model built from a monoclinic (*m*-) distortion of *Pb*am

with $\gamma \neq 90^\circ$. Note, not all reflections could be indexed using a single unit cell. Rietveld refinement of the full SXRD patterns for $Li_{1.6}Nb_2O_5$ and $Li_{1.2}Nb_2O_5$ lead to a decent fit with some minor discrepancies on the relative intensities. Optimization of the atomic positions was not performed due to the large number Nb atoms, 8 and 16 Nb positions in the orthorhombic and monoclinic model respectively, together with overlapping reflections. Moreover, an anisotropic broadening fitted with a strain model is observed with narrow (00l) and large (hk0) reflections for both phases.

2.3. Determination of tetragonal structures for powder *T*-Nb_2O_5

In Supplementary Fig. 13 and Table 2, the structural model for the tetragonal (*t*-) phase was determined as follow. Unit cell was found using Dicvol. Space group was chosen to be *P4/mmm*, the highest symmetry space group. Nb and O positions were determined from simulated annealing as implemented in Fullprof, and Nb occupancies were refined leading to a Nb/O ratio of 0.42. Nb-O average distance is 2.1 Å, which is just slightly larger than the average Nb-O distance in NbO_2, 2.05 Å, (Nb^{4+}) suggesting a lower Nb oxidation state in this compound ($n<4$, with Nb^{n+}), consistant with its formation at low voltage. Due to the low scattering power of Li, the presence of Li in the structure is unknown. However, based on the Nb/O and the suggested oxidation state, we hypothesize the presence of Li. Completely filling the remaining octahedral sites with Li would lead to the composition $Li_{1.15}Nb_{0.85}O_2$ with a Nb average oxidation state of +3.35 which is possible, although further investigations are necessary to test this hypothesis.

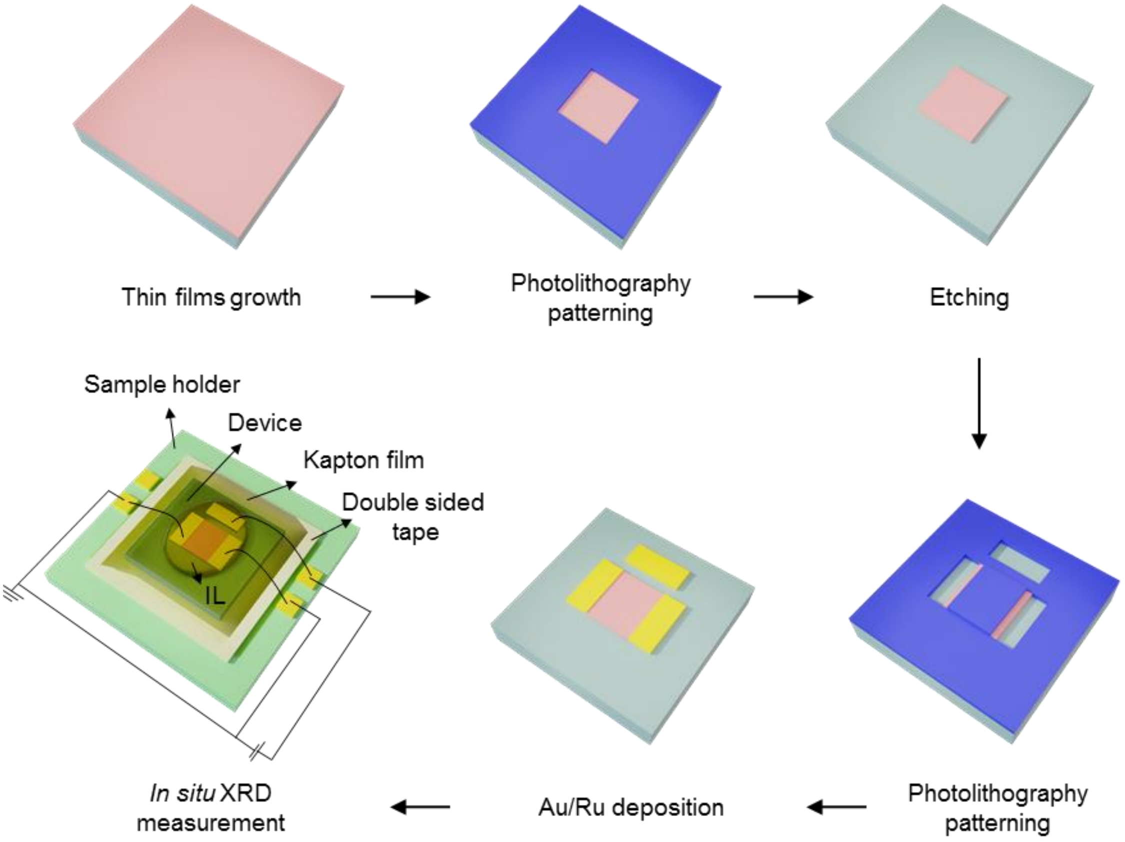

Supplementary Fig. 7. Schematics of device fabrications for *in situ* thin film XRD. The *T*-Nb_2O_5 channel with a size of 2 × 2 mm^2 was etched, then Au (70nm)/Ru (5 nm) layers were deposited for the gate and contact electrodes. The device was attached using double-side tape on a specially designed sample holder. The IL was placed on the device surface, and then Kapton film is attached to thin the IL thickness.

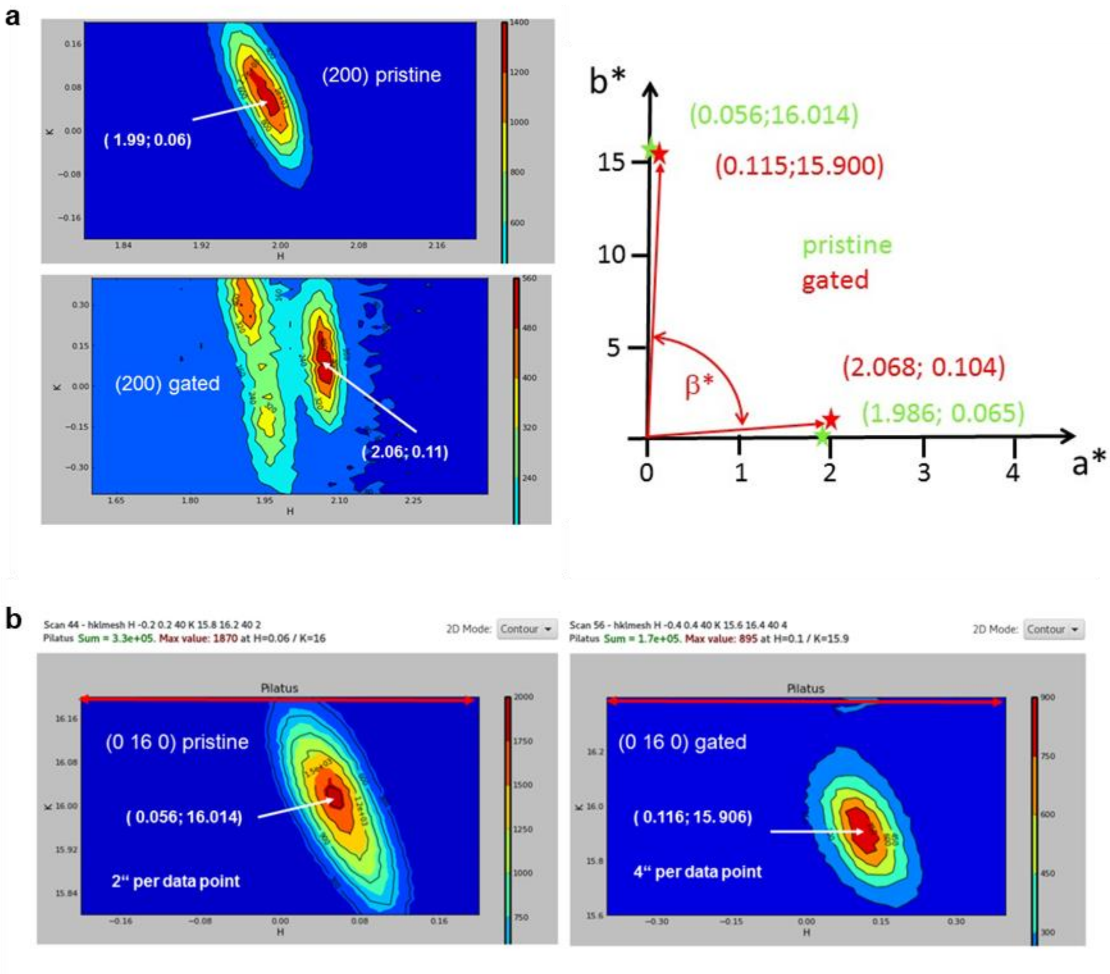

Supplementary Fig. 8. Reciprocal space mappings (RSM) of 100 nm thick *T*-Nb_2O_5/LSAT(110) using GaJet X-Ray source (λ=1.3414 Å) XRD. RSM of the pristine and gates samples for the **a,** (200) and **b,** (0<u>16</u>0) reflections. The positions of the peaks are indicated. The inset shows a schematic of the reflection positions for the pristine (p) and the gated (g) sample. After gating, the scattering vectors of the (200) and the (0<u>16</u>0) reflection are rotated by +0.7 and -2.6 degrees indicating a relative modification of the (reciprocal) angle γ* by 3.3 degrees.

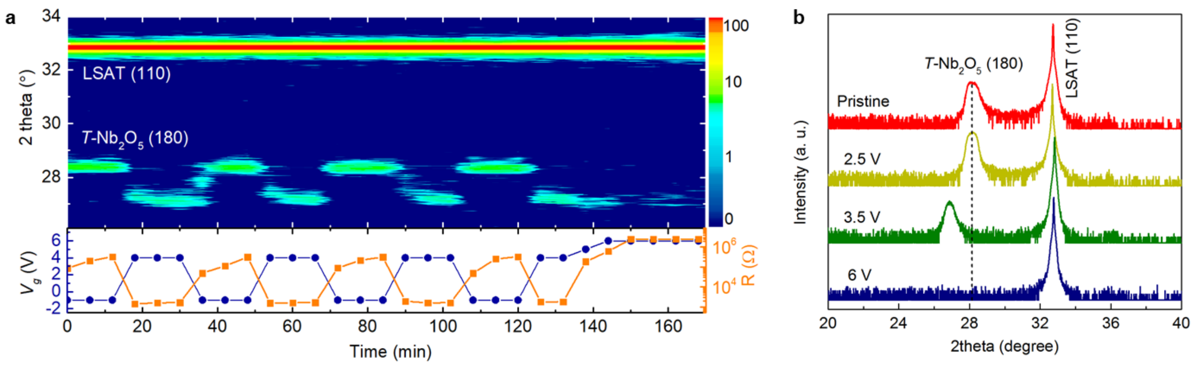

Supplementary Fig. 9. *In situ* and *ex situ* XRD via Li-ionic liquid gating. a, The *in situ* XRD measurements during Li-ionic liquid gating. The reversible structural and electronic property changes between the orthorhombic insulator and monoclinic metal are observed. The structure is collapsed after 6 V gating. 50 nm thick *T*-Nb_2O_5/LSAT (110) device is used. **B**, *ex situ* XRD with different gate voltage. 100 nm thick *T*-Nb_2O_5/LSAT (110) film is gated. The 2.5 V gated film does not show structural changes, while 3.5 V gated film shows the (180) peak shift, indicating a transition from orthorhombic to monoclinic phase. The 6 V gated film show the disappearance of the *T*-Nb_2O_5 (180) peak.

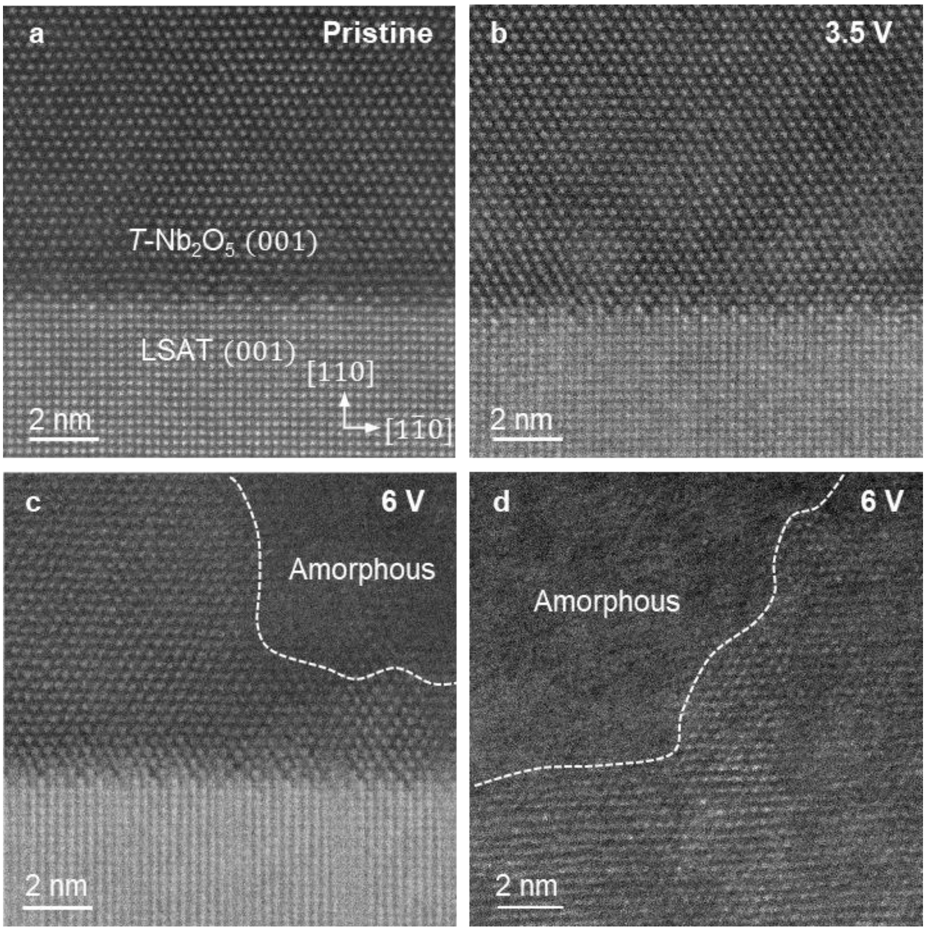

Supplementary Fig. 10. Cross-sectional *ex situ* STEM-HAADF images of pristine and gated *T*-Nb_2O_5/LSAT(110). a, pristine, **b**, 3.5 V, and **c, d** 6 V gated films. The 3.5 V gated film does not show noticeable changes of structures from the STEM image, while the 6 V gated film shows partially amorphous structures. The film thicknesses are 30 nm.

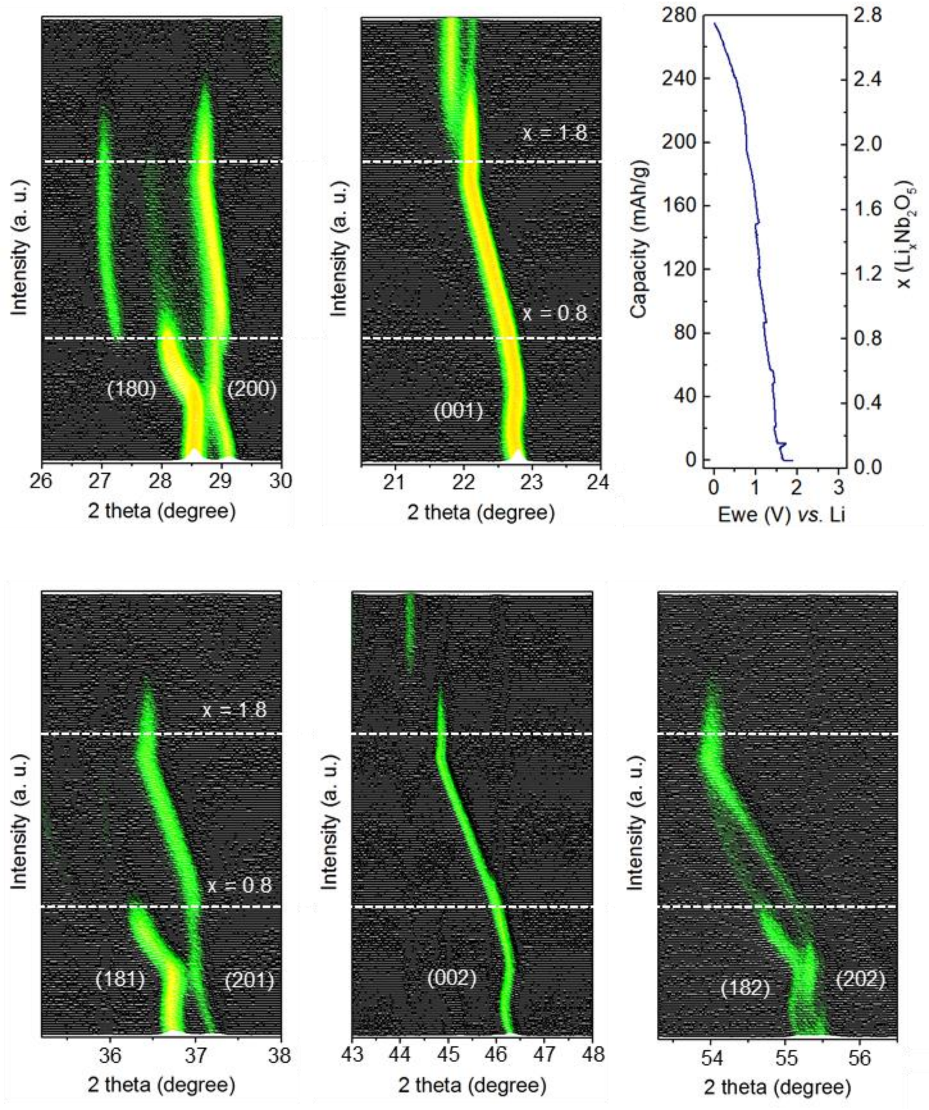

Supplementary Fig. 11. ***In situ*** **XRD data for bulk powder *T*-Nb_2O_5.** Monoclinic distortion starts at x = ≈0.8 (x in $Li_xNb_2O_5$), and the tetragonal structure shows from x = ≈1.8. The measurement was done until the deep discharge potential of 0.005 V vs Li with C/34 scan rate for electrochemical cycling. The upper right panel shows the corresponding discharge curve.

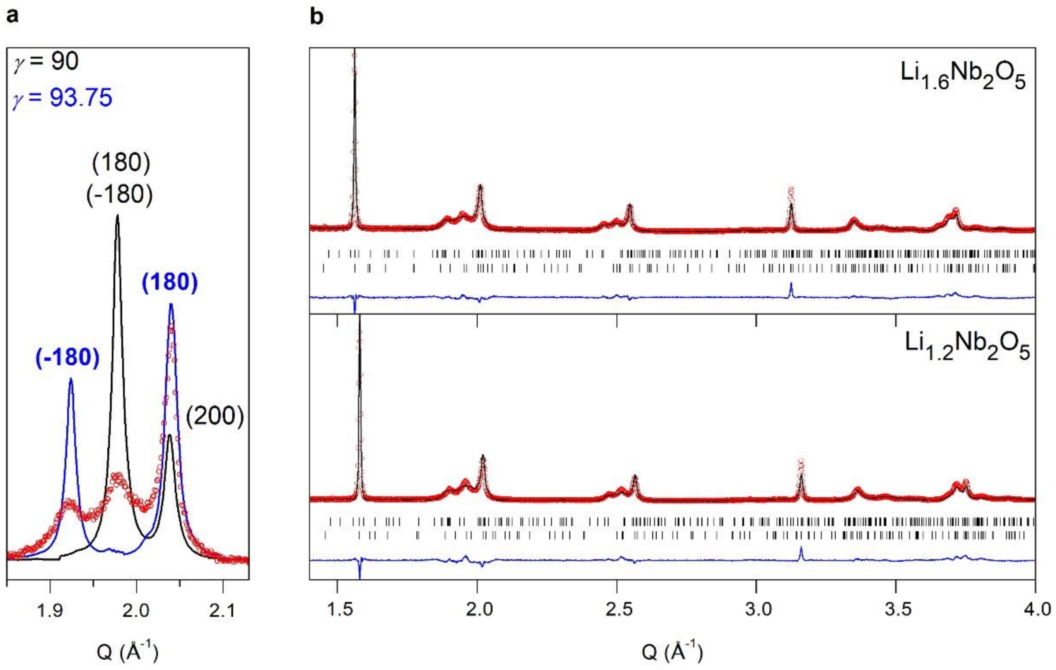

Supplementary Fig. 12. a, SXRD patterns of *ex situ* $Li_{1.6}Nb_2O_5$ together with simulated patterns of the *Pbam* and *P2/m*, in black and blue, respectively. **b**, SXRD patterns and Rietveld refinements of $Li_{1.2}Nb_2O_5$ and $Li_{1.6}Nb_2O_5$. Red circles, black line and blue line represent the observed, calculated and difference patterns, respectively.

Supplementary Table 1. Crystallographic table for the monoclinic phase in the SXRD pattern of $Li_{1.6}Nb_2O_5$. Li positions are omitted because unknown due to the low scattering power of Li compared to Nb and O.

***m*-$Li_{1.6}Nb_2O_5$ (*P2/m*): *a* = 6.262(4) Å, *b* = 30.074(12) Å, *c* = 4.0180(4) Å, *γ* = 94.13(2) °**

	x	y	z	Occ	Biso (Å²)	multiplicity
**O1_1**	0.2823	0.0388	0	1	1	2
**O1_2**	0.7823	0.4612	0	1	1	2
**Nb2_1**	0.1857	0.1537	0.5477	1	0.5	4
**Nb2_2**	0.6857	0.3463	0.4523	1	0.5	4
**Nb3_1**	0.2522	0.27586	0.547	1	0.5	4
**Nb3_2**	0.7522	0.22414	0.453	1	0.5	4
**Nb4_1**	0.2376	0.40702	0.5543	1	0.5	4
**Nb4_2**	0.7376	0.09298	0.4457	1	0.5	4
**Nb5_1**	0.054	0.338	0	1	0.08	2
**Nb5_2**	0.554	0.162	0	1	0.08	2
**Nb6_1**	0.434	0.4776	0	1	0.08	2
**Nb6_2**	-0.066	0.0224	0	1	0.08	2
**Nb7_1**	0.435	0.213	0	1	0.04	2
**Nb7_2**	-0.065	0.287	0	1	0.04	2
**Nb1_1**	0.2807	0.03551	0.5445	1	0.5	4
**Nb1_2**	0.7807	0.46449	0.4555	1	0.5	4
**O1_1**	0.2823	0.0388	0	1	1	2
**O1_2**	0.7823	0.4612	0	1	1	2
**O2_1**	0.19	0.1562	0	1	1	2
**O2_2**	0.69	0.3438	0	1	1	2
**O3_1**	0.246	0.2782	0	1	1	2
**O3_2**	0.746	0.2218	0	1	1	2
**O4_1**	0.262	0.414	0	1	1	2
**O4_2**	0.762	0.086	0	1	1	2
**O5_1**	0.063	0.0879	0.5	1	1	2
**O5_2**	0.563	0.4121	0.5	1	1	2
**O6_1**	0.07	0.2199	0.5	1	1	2
**O6_2**	0.57	0.2801	0.5	1	1	2
**O7_1**	0.3423	0.3441	0.5	1	1	2
**O7_2**	0.8423	0.1559	0.5	1	1	2
**O8_1**	0.1166	0.4688	0.5	1	1	2
**O8_2**	0.6166	0.0312	0.5	1	1	2
**O9_1**	0.436	0.106	0.5	1	1	2
**O9_2**	-0.064	0.394	0.5	1	1	2
**O10_1**	0.456	0.1964	0.5	1	1	2
**O10_2**	-0.044	0.3036	0.5	1	1	2
**O11_1**	0	0	0.5	1	0.25	1
**O11_2**	0.5	0.5	0.5	1	0.25	1

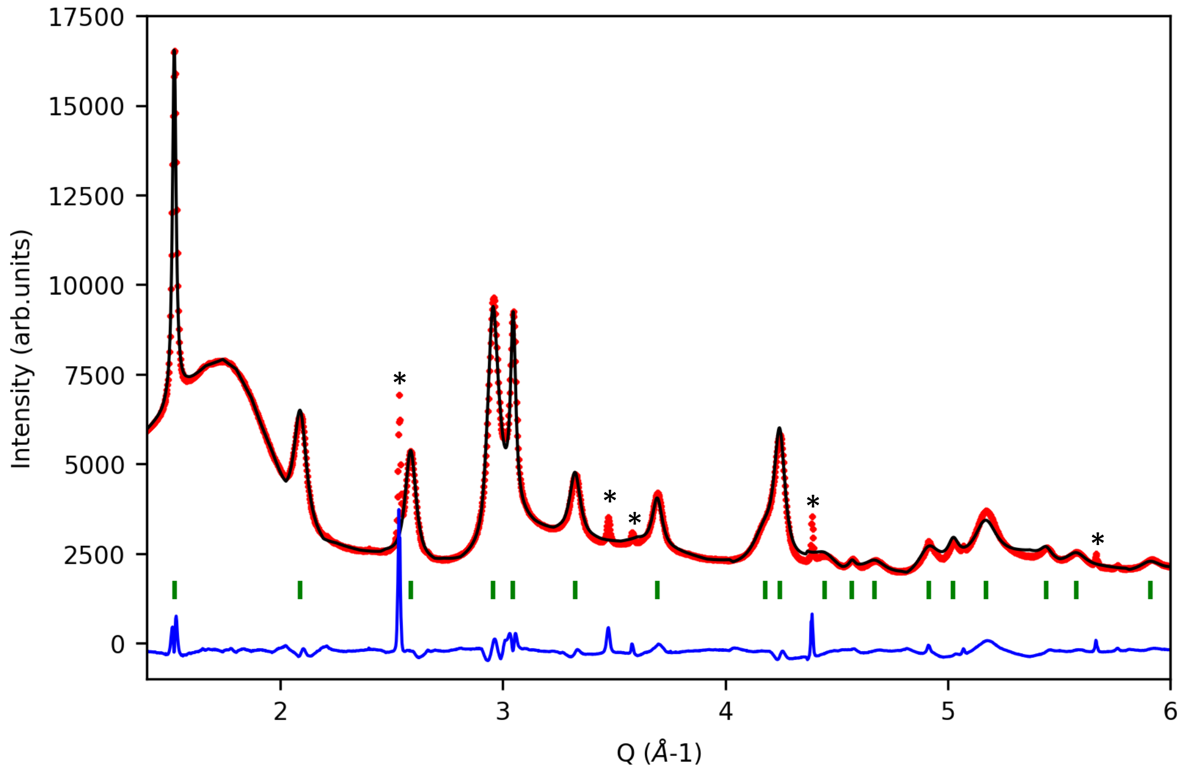

Supplementary Fig. 13. Rietveld refinement of an *operando* SXRD pattern done at 5 mV with the tetragonal phase described in Supplementary Table 1. The *operando* cell produces a large background together with peaks marked *. Red circles, black line and blue line represent the observed, calculated and difference patterns, respectively. Green vertical bars represent the Bragg position

Supplementary Table 2. Crystallographic table for the *t*-$Li_{2.5}Nb_2O_5$. Possible Li positions are omitted because unknown due to the low scattering power of Li compared to Nb and O.

***t*-$Li_{2.5}Nb_2O_5$ (*P4/mmm*): *a* = 3.0073(2) Å, *c* = 4.1249(3) Å**

	x	y	z	Occ	Biso (Å²)	multiplicity
**O1**	0	0	½	1	1	4
**O2**	½	½	0	1	1	4
**Nb1**	0	0	0	0.69(2)	1	4
**Nb2**	½	½	½	0.151(2)	1	4

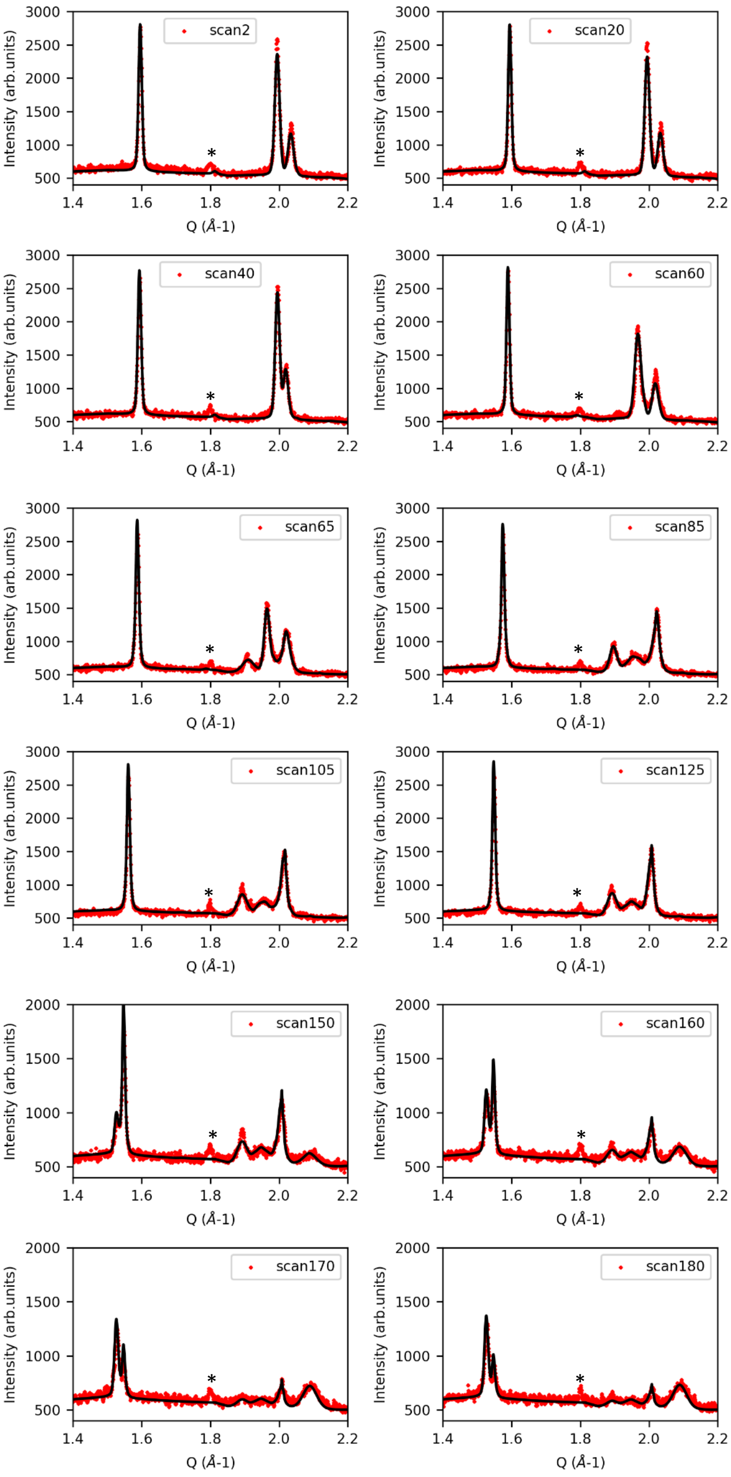

Supplementary Fig. 14. Rietveld refinements of the *operando* XRD on powder *T*-Nb_2O_5. Scans 2-60 are refined with the orthorhombic model only. Scans 65-125 are refined with the orthorhombic and monoclinic models. Scans 150-180 are refined all models (orthorhombic, monoclinic and tetragonal). * comes from the sample holder. Red crosses and black line represent the observed and calculated patterns corrected form the shift due to the sample height, respectively.

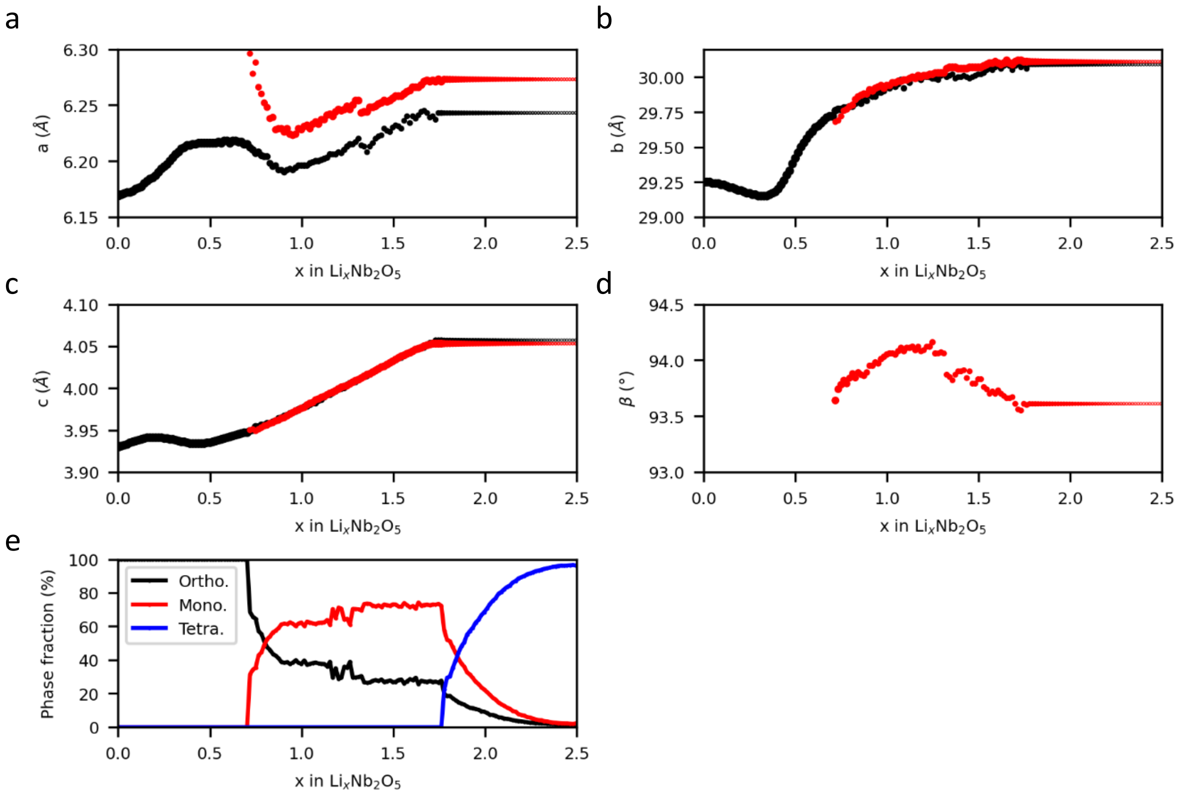

Supplementary Fig. 15. a-d, Cell parameters obtained from the Rietveld refinement for the orthorhombic and monoclinic phase in black and red, respectively. The marker size is proportional to the phase fraction. **e**, phase fraction obtained from the Rietveld refinement. X in $Li_xNb_2O_5$ is determined from the applied current and active material mass used during the *operando* XRD experiment.

3. Computational methods and results

3.1. Computational methods

First-principles calculations use density functional theory with a plane-wave basis set, as implemented in the QUANTUM ESPRESSO (QE) package[2]. Ultrasoft pseudopotentials[3] along with Perdew, Burke, Ernzerhof (PBE) exchange correlation (XC)[4] functional were used. Dispersion interactions are accounted for using the Grimme DFT-D3 method[5]. Phonon calculations are performed using the DFT-D2 scheme due to technical computational considerations in performing this type of calculations using QE. The DFT+*U* method[6-8], with *U*=6.0 eV, is applied to account for d-electron localization on the Nb atoms[9]. The plane wave kinetic-energy cutoff for wave functions is set at 40 Ry, with the cutoff for charge density being ten times larger. The Brillouin zone is sampled using a 6×1×8 Monkhorst-Pack mesh[10] for the unit cell of un-lithiated [T-Nb_2O_5] ($Li_4Nb_{16}O_{42}$ in our model, see the introduction in Supplementary Section 3.2). The $2a \times b \times 2c$ and $a \times b \times 3c$ super cells of T-Nb_2O_5 are sampled with a 3×1×4 k-point mesh. We modeled the diffusion of one Li-ion in the unit cell of T-Nb_2O_5 by the nudged elastic band method with climbing image (CI-NEB)[11,12]. The free-energy profile of the most favorable diffusion pathway has been shown in Fig. 3g.

3.2. Introduction of our *T*-Nb_2O_5 simulation model

The conventional unit cell of T-Nb_2O_5 material (with a space group of *Pbam*, orthorhombic crystal system, shown in Supplementary Fig. 16a), is composed of 16.8 Nb and 42 O atoms in two layers. All the Nb sites are fractionally occupied, with 16 Nb atoms in 4h layer located at four 8i Wyckoff positions with half occupancy-corresponding to pairs of sites just above and below the 4h plane (Supplementary Fig. 16c). The remaining 0.8 Nb atoms per unit cell are randomly scattered into four sets of three different Wyckoff positions in the 4g layer with occupancies of 0.08, 0.08, and 0.04 (Supplementary Fig. 16b). The Nb atoms in the 4h layer coordinate into NbO_6 units (distorted octahedral) or NbO_7 units (pentagonal bipyramidal). Because of the disordered positions due to fractional occupancy, the computational treatment on the conventional unit cell of T-Nb_2O_5 structure is performed as follows: the pairs of sites in the 4h layer were consolidated into 16 Nb atoms in the 4h plane and allowed to relax, while the 0.8 Nb in the 4g layer were replaced by 4 Li atoms distributed in this layer for charge compensation. Thus, we provide a new simulation model of $Li_4Nb_{16}O_{64}$ (Fig. 3b) which we denote as [T-Nb_2O_5] model with the relaxed lattice parameters as shown. Through the density of states (DOS) analysis (Supplementary Fig. 17b), our simulation model (Fig. 3c) shows a larger band gap (2.3 eV) than that (1.8 eV) of the previous theoretical model (eliminating 0.8

Nb in the 4g layer and charge compensating with $2V_O$ in the 4h layer; $Nb_{16}O_{40}$[13,14] calculated with the same method. In comparison, our simulation model shows a better gap estimate, closer to the experimental band gap of 3.6 eV.

3.3. Determination of most favorable O sites for the external Li intercalation

Here, we investigated the activity of different O sites to adsorb one extra Li on their top sites in [T-Nb_2O_5]. As shown in Supplementary Fig. 18, T-Nb_2O_5 consists of two distinct layers (4g layer and 4h layer, Supplementary Fig. 18a. As for the 4h layer, it is a hybrid Nb-O layer, in which oxygen atoms, which may have two distinct coordinations (O_{2c} and O_{3c}, Supplementary Fig. 18b), may be active sites that can adsorb more Li on top of it. O_{3c} atoms are coordinated by three neighboring Nb atoms, which belong to two NbO_7 and one NbO_6 units, while O_{2c} atoms are coordinated by two neighboring Nb atoms, which belong to one NbO_7 and one NbO_6. The bridge and hollow sites of O_{2c} and O_{3c} are found unfavourable, because Li will spontaneously transfer to the top site of either O_{2c} or O_{3c}. In the case of these two candidates, Li intercalates into the 4g layer, on top of O_{2c} and O_{3c} sites (in 4h layer). The top views of these two structures (after relaxation of ion positions and lattice parameters) are shown in Supplementary Fig. 18c and 18e, respectively. Furthermore, we also considered another Li intercalation configuration, where Li is located in the 4h layer together with Nb cations (Supplementary Fig. 18d).

The O candidates in the 4g layer (Fig. 3b) are inactive based on the calculated ΔE_b, because they are vertically coordinated by Nb atoms in 4h layer. Moreover, we found it can be spontaneously relaxed back to the ground-state configuration (describe later) without any barrier. As for O candidates in the 4h layer, they can be categorized into two different kinds: O_{3c} and O_{2c} as discussed above (Supplementary Fig. 18). The first extra Li prefers to be adsorbed on the top site of O_{2c} with a larger binding energy (ΔE_b = -2.10 eV, defined in Methods) than that of O_{3c} (ΔE_b = -2.03 eV) and other less favorable O sites. Therefore, we found the top sites of O_{2c} provides the ground-state location (with strongest binding energy) for Li intercalation. Once O_{2c} sites are fully occupied, more Li will start to be adsorbed on the top of less favorable O_{3c} sites. Based on this, we determined the active sites (coordination environment) by computing the differential binding energy for each Li intercalation of Li_x-[T-Nb_2O_5] (x = 0.12 ~ 1.08) as shown in Supplementary Fig. 19. We note that in this step, many trial sites have nearly identical binding energies. This, when coupled with the low barriers for Li diffusion suggests that there are actually an ensemble of structures with slightly different Li distributions, and our calculations identify one reasonable representative. This is consistent with previous

findings from NMR showing that Li occupies a distribution of similar, but not identical, sites[15]. The configuration with the lowest energy is selected for the further analysis and is used as precursor for the next Li intercalation. The most favorable site identified by DFT for each Li interstitial (in the [*T*-Nb_2O_5] unit cell) is summarized in Supplementary Fig. 20. Moreover, it has been well-established (including with the XRD data presented in this manuscript) that intercalation of small amounts of Li causes an expansion in the cell volume without substantially changing the overall Nb_2O_5 structure[16]. This finding and the observation of fast, reversible Li movement suggests that Li is located in the relatively open 4g layer, which is supported both by Raman experiments[13] and our DFT calculations.

3.4. The onset Li concentration for conductivity in two kinds of super cells and unit cell

To model low concentrations of Li intercalation and avoid spurious interactions between periodic images in the smallest c direction in the unit cell, we created two kinds of super cells (2a × b × 2c and a × b × 3c) of *T*-Nb_2O_5 with 248 and 186 atoms in the model, respectively. The (a × b × 3c) super cell is the smallest model to observe the new valence band (induced by one extra Li interstitial) wholly below the Fermi energy as shown in Fig. 3d and Supplementary Fig. 17b, while unit cell and (2a × b × 2c) super cell are directly tuned to metal once even one extra Li atom inserted into the models. The above findings in three models indicate that the accurate DFT-estimation of the onset of conductivity requires a very large simulation model, especially in c direction. Therefore, our findings in the (a × b × 3c) super cell have already been computationally meaningful to unveil the electronic evolution properties of *T*-Nb_2O_5 affected by extra Li interstitial (see Figure 3 and related discussions in the main texts).

3.5. Appendix structural files corresponding to Figure 3

All fully relaxed configurations of Li_x-[*T*-Nb_2O_5] we study in this paper are provided as Supporting Data. Detailed description of the structures we used for the results in Figure 3 (shown in main text):

- Figure 3(b) and (c), the structure can be found in the folder named “Unit Cell” .
- Figure 3(d) and (e), the structures can be found in the folder named “Super Cell 33_Li_x” with the file names “Li0.04_T_Nb2O5.xsf” and “Li0.08_T_Nb2O5.xsf”.
- Figure 3(f), the structure can be found in the folder named “Unit Cell_Li_x” with the file name “Li1.08_T_Nb2O5.xsf”
- Figure 3(g), the structure can be found in the folder named “Unit Cell_Li_x” with the file name “Li0.12_T_Nb2O5.xsf”

- Figure 3(h), the structures can be found in the folders named “Unit Cell_Li_x” and “Super Cell 22_Li_x”, all files are related.
- Figure 3(i), the structures can be found in the folder named “Monoclinic”, all files are related.

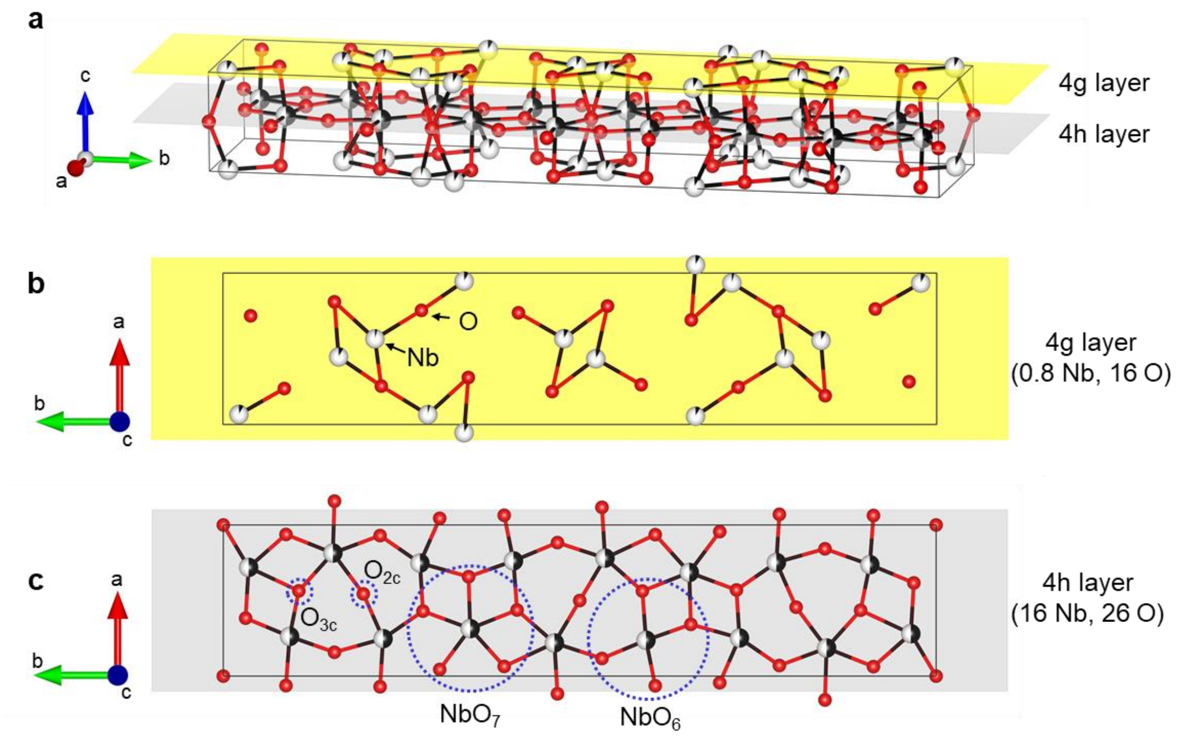

Supplementary Fig. 16. **a,** The front view of the conventional unit cell of *T*-Nb_2O_5 ($Nb_{16.8}O_{42}$), while top views of 4g layer and 4h layer are shown in **b** and **c**, respectively.

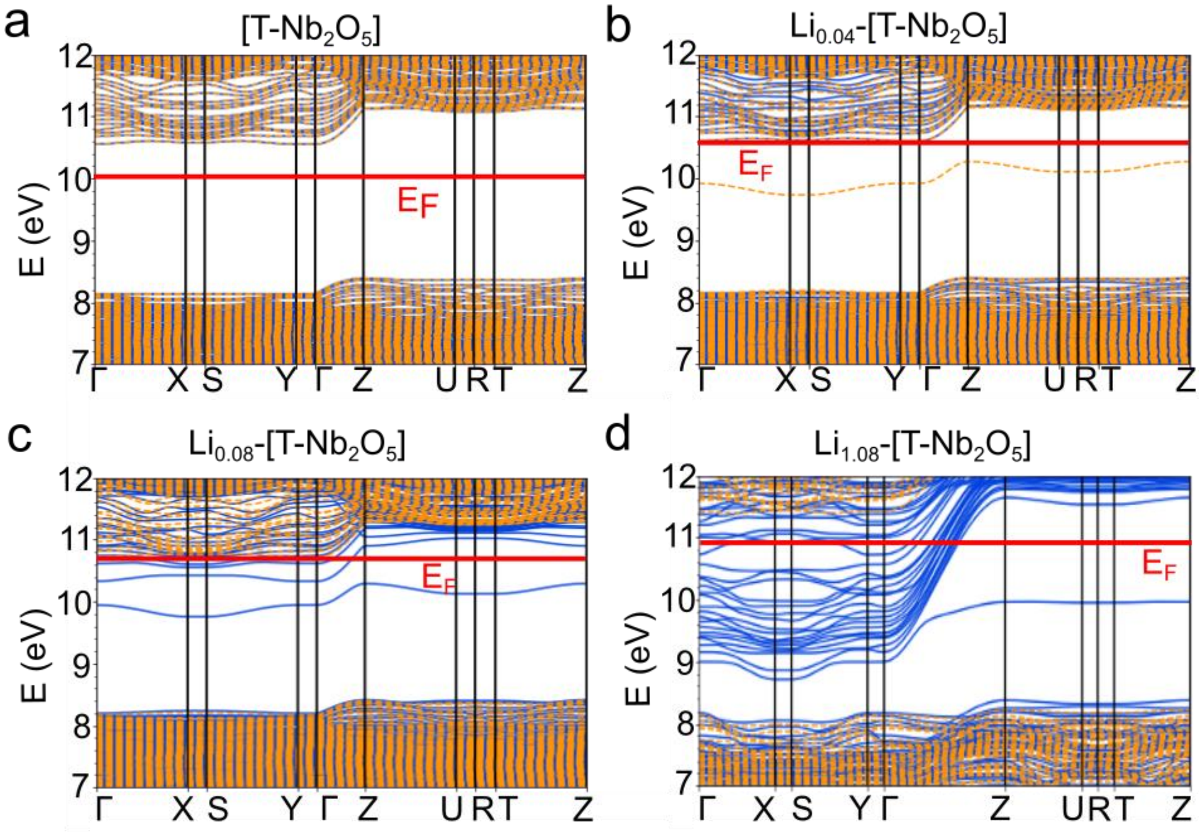

Supplementary Fig. 17. **a-c**, Band structures of the (a × b × 3c) [*T*-Nb_2O_5] super cell with 0, 1, and 2 extra Li interstitial. **d,** Band structure of the $Li_9Nb_{16.8}O_{42}$ model in monoclinic phase.

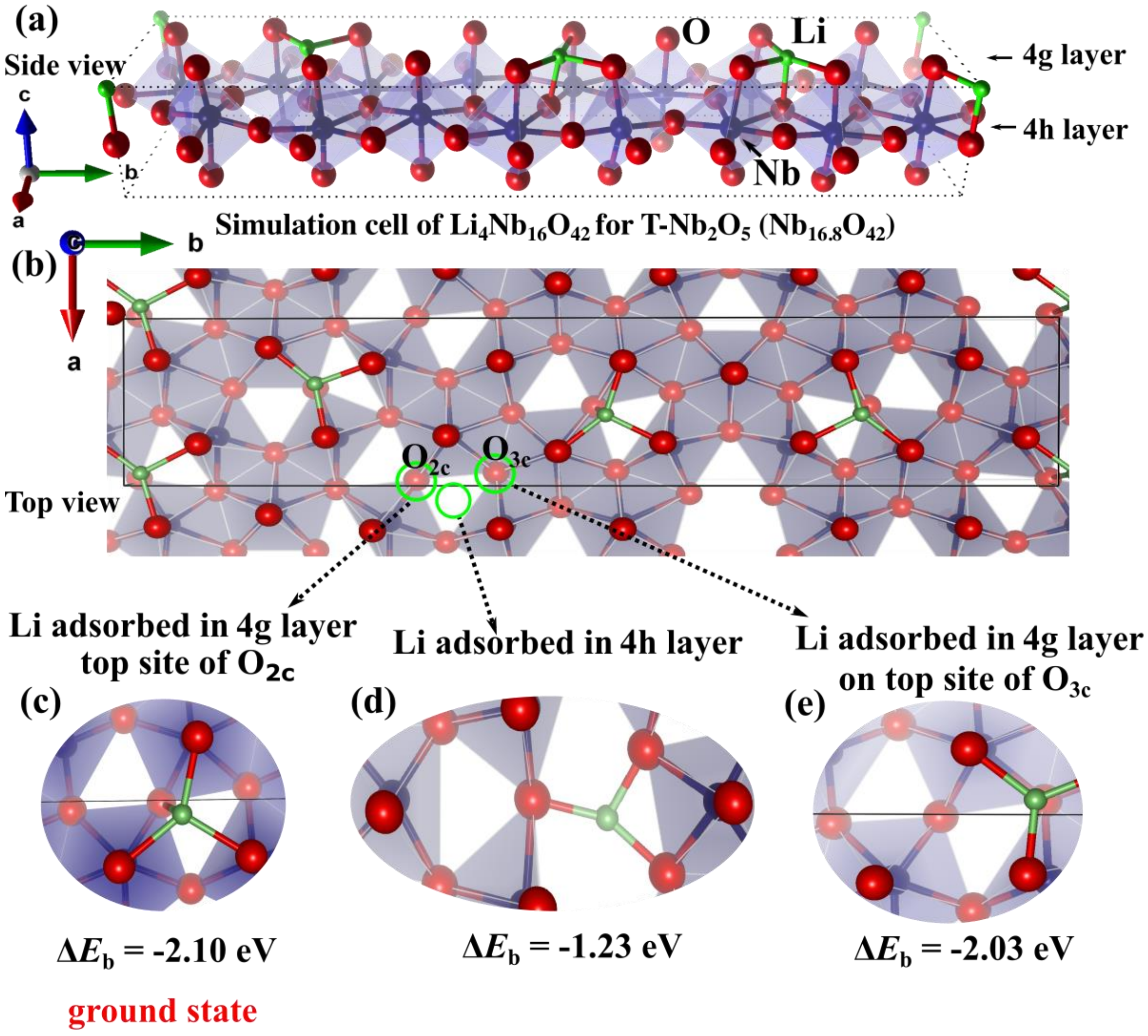

Supplementary Fig. 18. **(a)** and **(b)** show the side and top views, respectively, of the orthorhombic model system ($Li_4Nb_{16}O_{42}$) used to simulate the conventional unlithiated unit cell of *T*-Nb_2O_5 ($Nb_{16.8}O_{42}$). O2c and O3c represent the O ions in the 4h layer coordinated by two and three adjacent Nb ions in the same plane (4h layer). **(c)-(e)** Three possible configurations of an extra Li^+/e^- intercalation in *T*-Nb_2O_5, where Li^+/e^- intercalates the 4g layer in (c) and (e) (on top of O_{2c} and O_{3c} sites, respectively) and Li^+/e^- is in the 4h layer (in panel (d)).

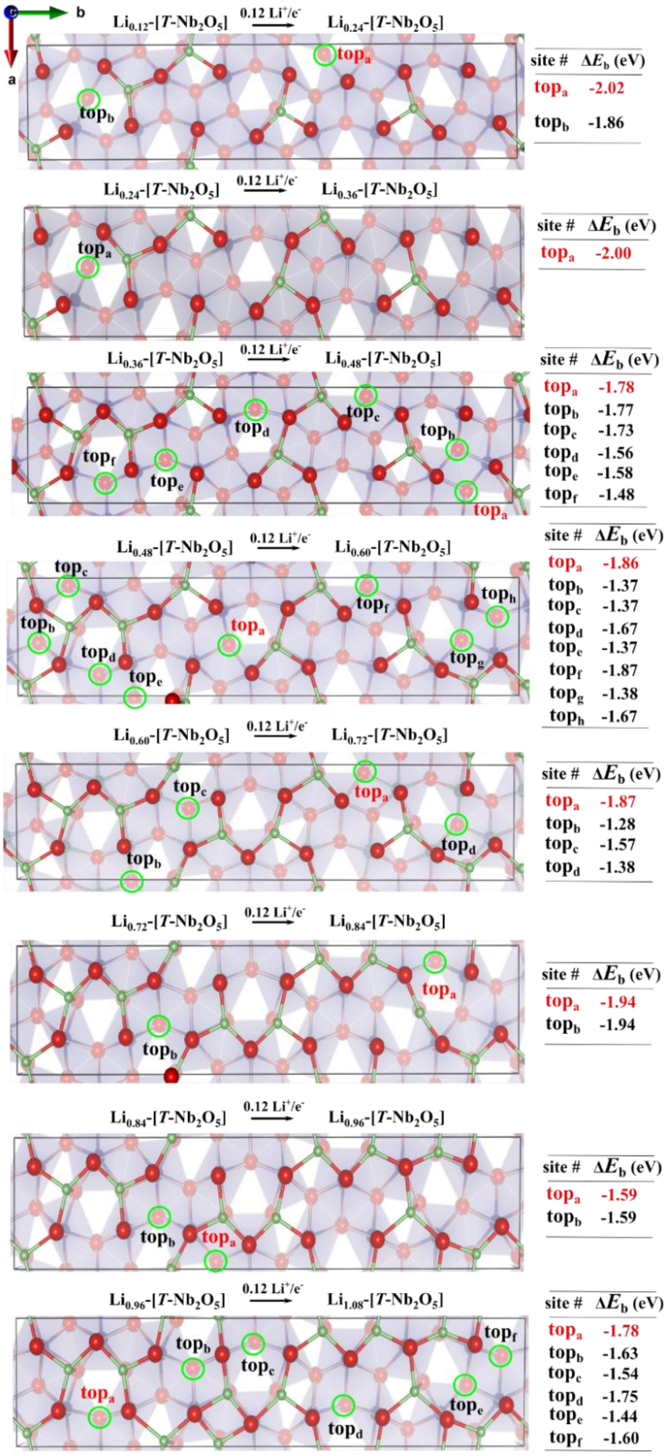

Supplementary Fig. 19. Top view of active sites associated with differential binding energies for each Li^+/e^- intercalation of Li_x-[T-Nb_2O_5] (x = 0.12 ~ 1.08), where 0.12 Li^+/e^- indicates a pair of Li^+/e^- adsorbed in T-Nb_2O_5 (because we convert $Nb_{16.8}O_{42}$ to Nb_2O_5). Li^+/e^- pair intercalate in 4g layer and on top of the candidate sites as we shown in each configuration of Li_x-[T-Nb_2O_5], while the most favorable site (providing the strongest differential binding energy) for each Li^+/e^- is labeled by top_a highlighted in red. In the configuration of $Li_{0.72}$-[T-Nb_2O_5] and $Li_{0.84}$-[T-Nb_2O_5], both of two active-site candidates are favorable.

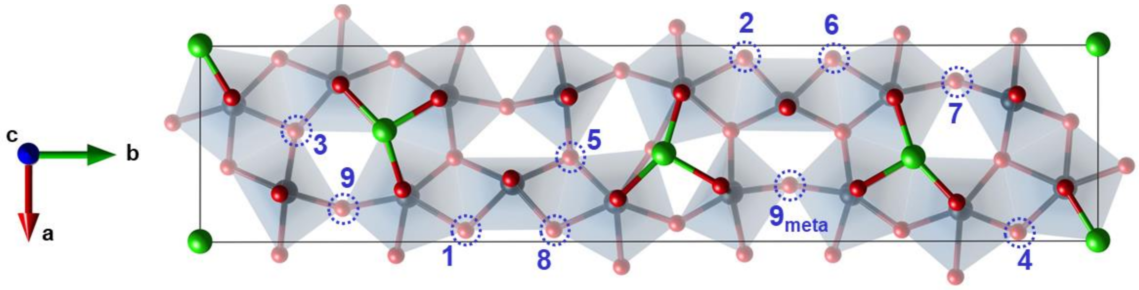

Supplementary Fig. 20. The most favorable sites for each Li interstitial (from 1st to 9th) in our *T*-Nb_2O_5 model (unit cell). The determination is based on our ΔE_b values when Li adsorbs atop the available O atoms in 4h layer. The site tagged with 9_{meta} is the metastable structure for $Li_{1.08}Nb_2O_5$, which is also favorable to be tilted into monoclinic phase (see Supplementary Fig. 21).

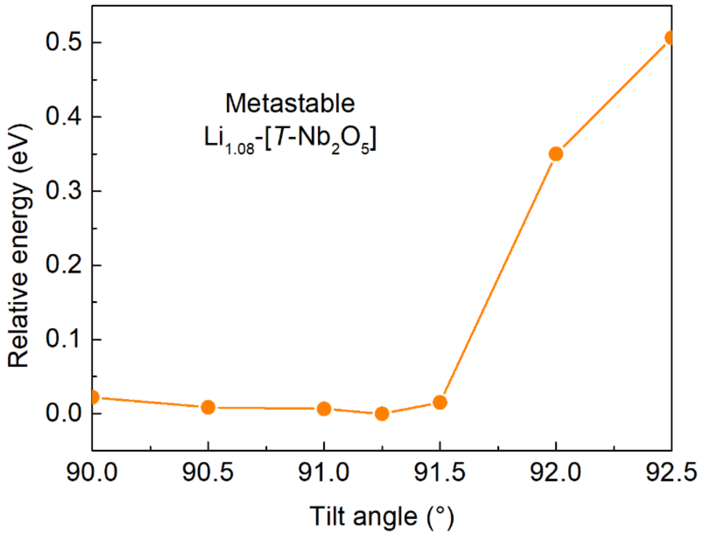

Supplementary Fig. 21. The relative energy evolution of the metastable structure $Li_{1.08}$-[*T*-Nb_2O_5] as a function of tilt angle, from 90° to 92.5°, where the Li from 1st to 8th are inserted at the most favorable sites shown in Supplementary Fig. 20, while the 9th Li is inserted at the 9_{meta} (less favorable O sites) site.

4. Electrochemical tests of *T*-Nb_2O_5

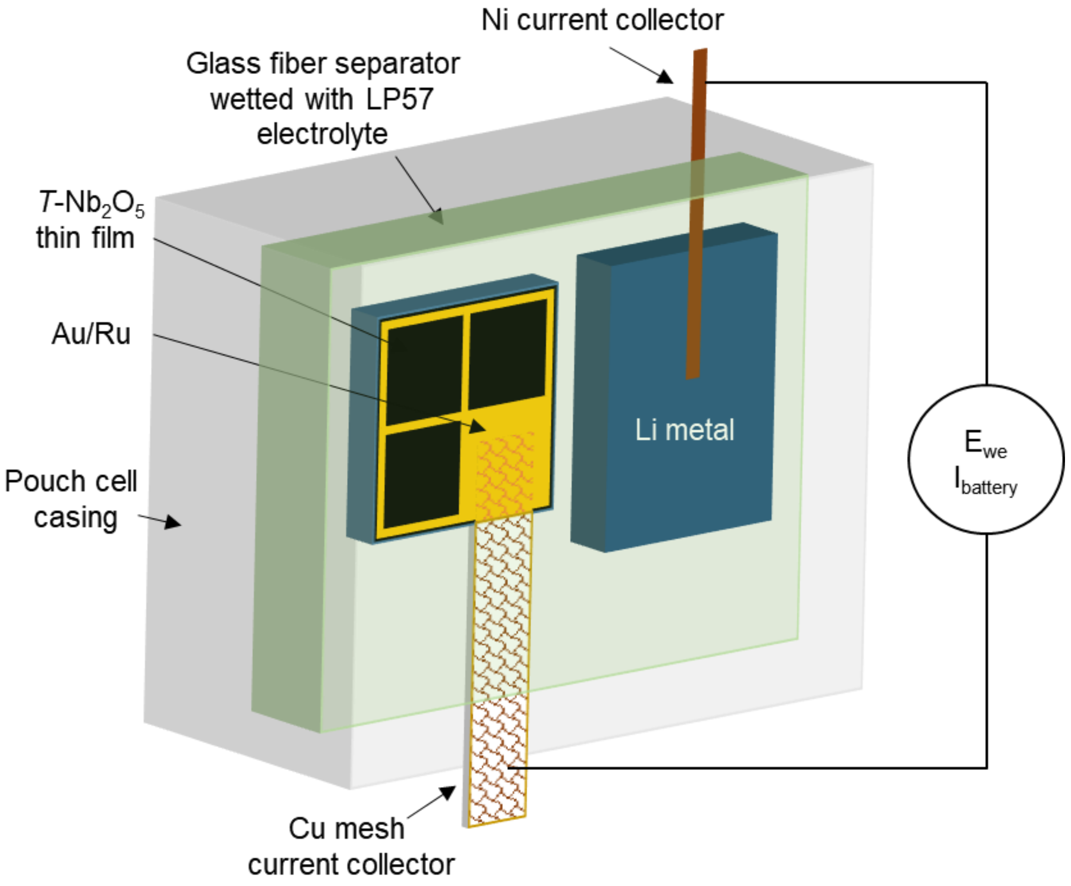

Supplementary Fig. 22. A schematic Pouch cell of *T*-Nb_2O_5 thin film for battery testing. The current, $I_{battery}$ and E_{we} is measured between *T*-Nb_2O_5 thin film and Li anode. By analogy with the gating experiment, *T*-Nb_2O_5 is the channel and Li metal is the gate electrode, hence the $I_{battery}$ and E_{we} correspond to the leakage current, I_g, and the gate voltage respectively. Note that the conventions are different: $I_{battery} = - I_g$.

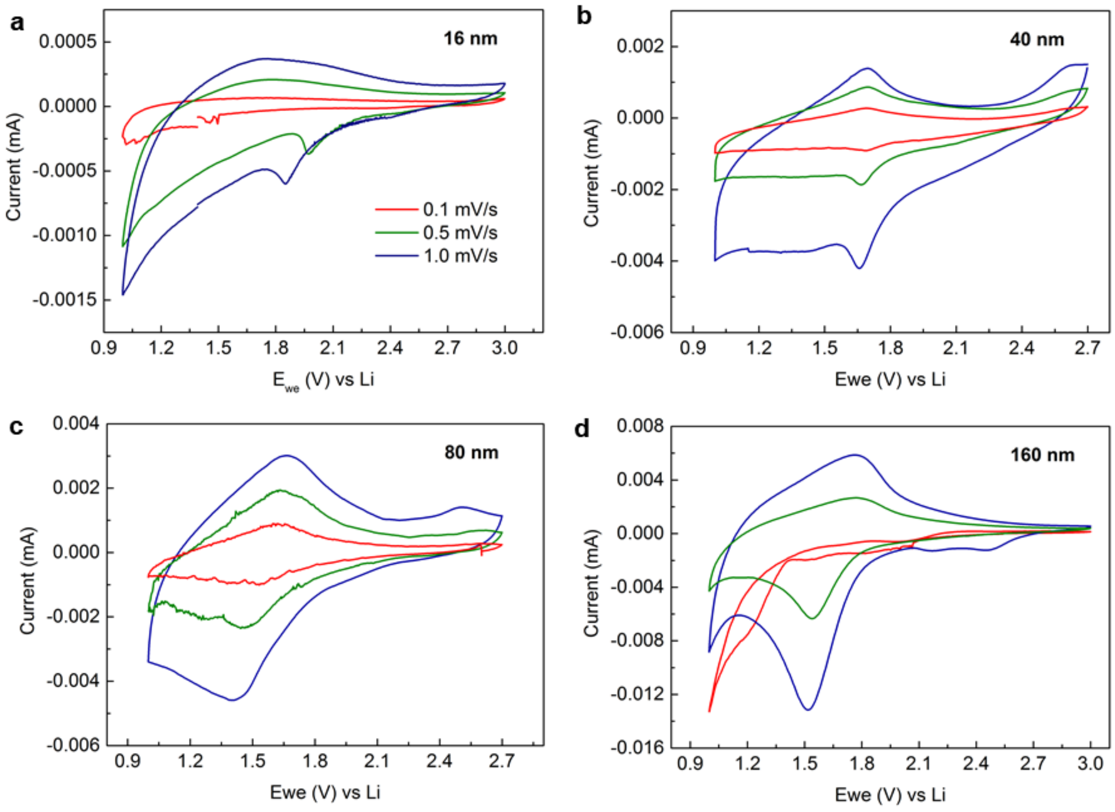

Supplementary Fig. 23. Cyclic voltammogram for *T*-Nb_2O_5/LSAT (110) thin films at different thicknesses such as **a**, 16 nm, **b**, 40 nm, **c**, 80 nm, and **d**, 160 nm. Cyclic voltammogram was recorded with different scan rate (0.1, 0.5 and 1.0 mV/s), and second cycle of each cycling rate is shown here. Irreversible reactions seem occurs at low voltages < 1 V as observed by the rise of the current.

5. Transport properties of T-Nb_2O_5 thin films via lithiation

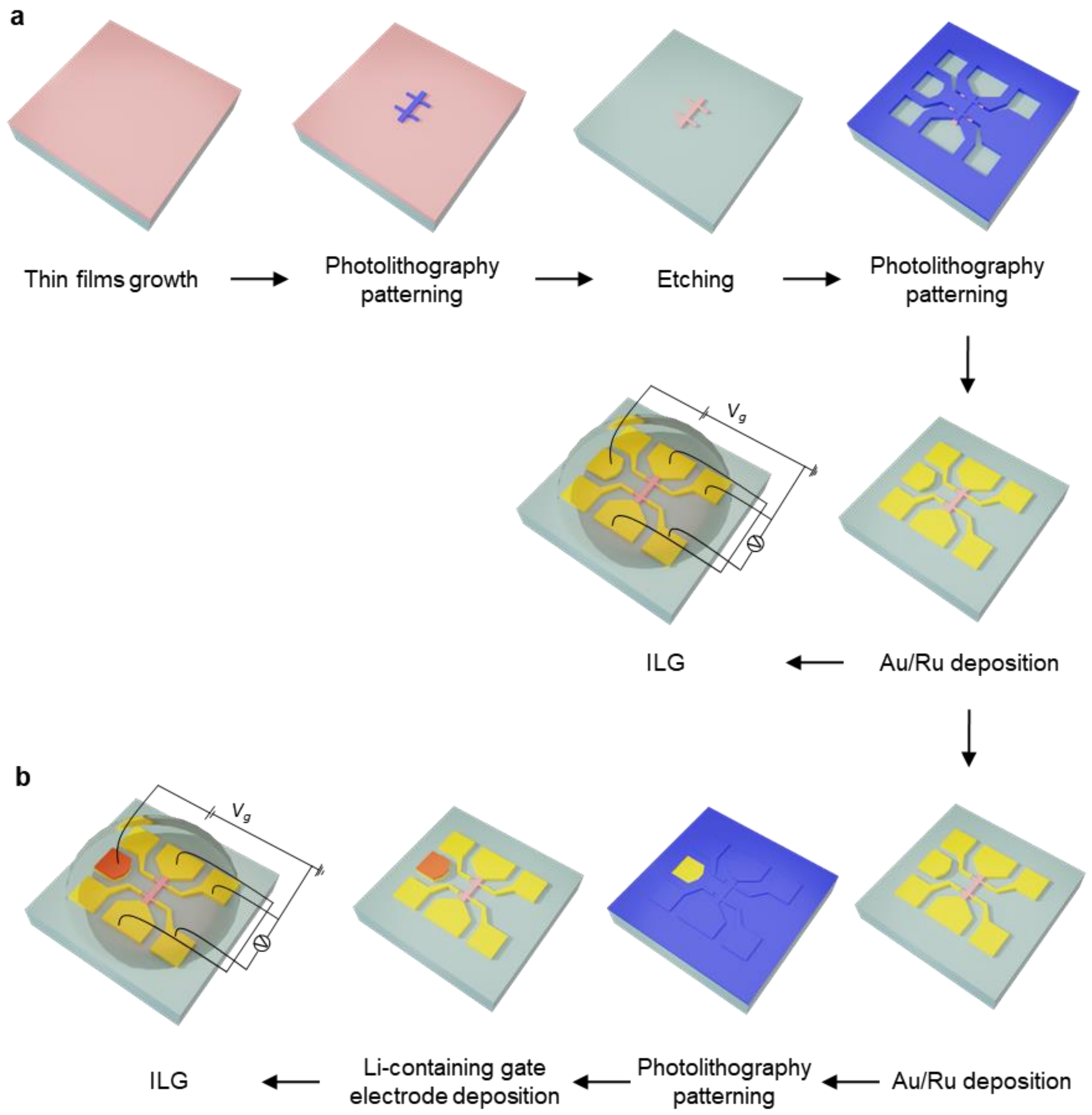

Supplementary Fig. 24. Schematic diagrams of device fabrications. a, Schematics of ionic liquid gating device fabrications. The standard photo-lithographic techniques are used. The channel was etched, and then Ru (5 nm) and Au (70 nm) are deposited successively for the gate electrode and channel contacts. The Li-ionic liquid was placed on the device for the gating experiment. **b**, Schematics of the device fabrications having Li-containing gate electrode. The Li-containing oxides are deposited on the Au/Ru gate electrode by pulsed laser deposition. The channel sizes are 65×30 μm^2.

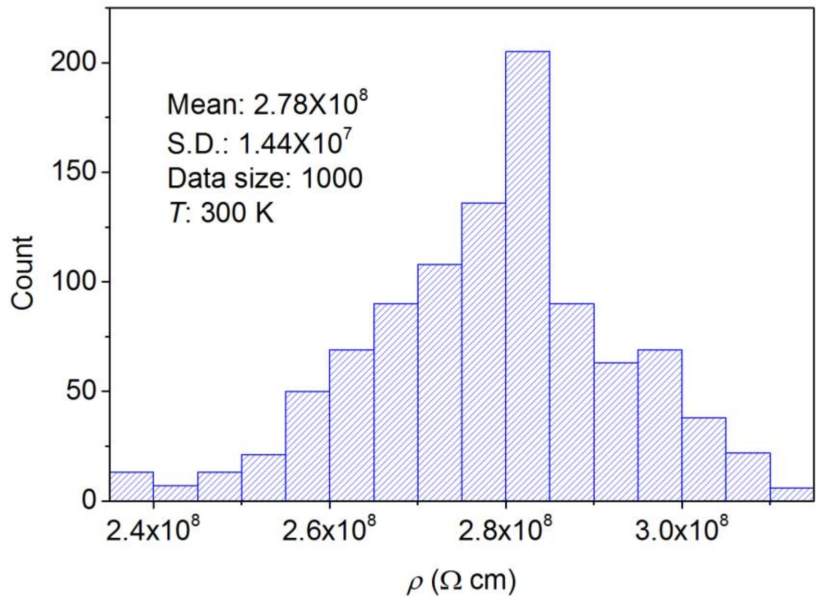

Supplementary Fig. 25. Resistivity measurements of the pristine *T*-Nb_2O_5/LSAT (110). The 1000 data points were averaged using a high resistance meter (B2985A, KEYSIGHT). During the measurement, the temperature of the sample was kept at 300 K in a vacuum environment.

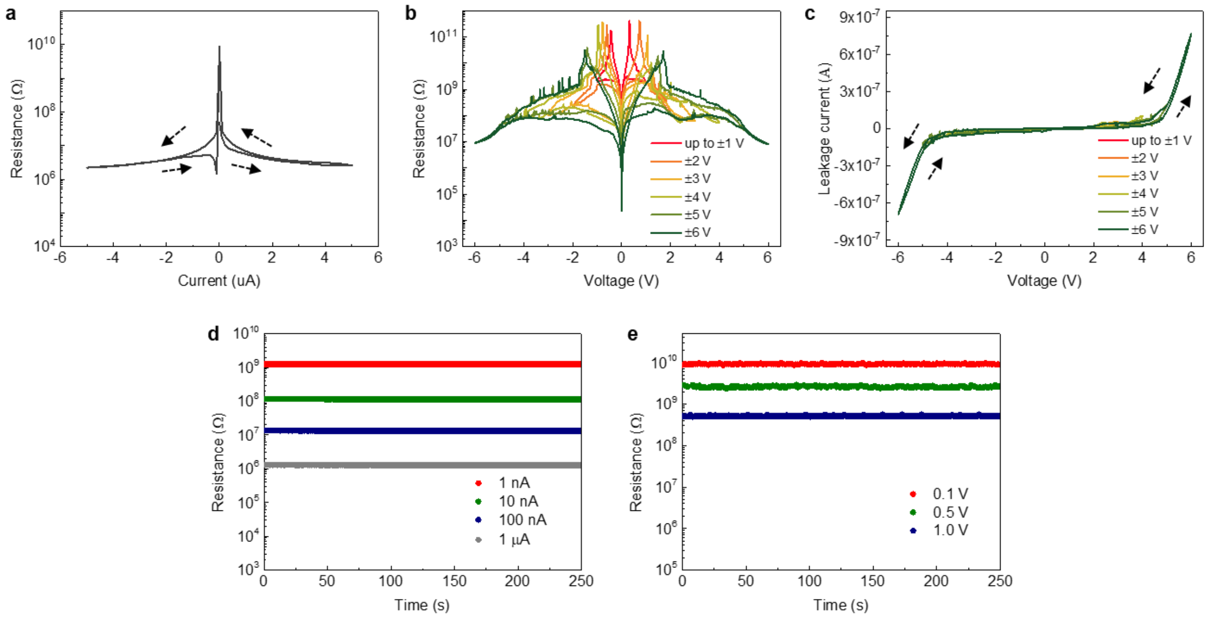

Supplementary Fig. 26. Source-drain current (I_{sd})- or voltage (V_{sd})-dependent Li-IL resistance. a, Resistance of Li-IL as a function of I_{sd}. **b**, Resistance and **c**, current of IL as a function of V_{sd}. Time-dependent changes in resistance of Li-IL at different **d**, I_{sd}- and **e**, V_{sd}. In order to measure the electrical resistance of ILs, we have placed the IL on a device made by LSAT (110) substrate, which does not interact via gating. The device has the same electrode structure (Fig. 2d) for ILG of *T*-Nb_2O_5 thin films for the comparison. The resistance of IL decreases from ≈10^{10} to ≈10^6 Ω when the applied current or voltage is increased and saturates at ≈10^6 Ω even upon application of high current (5 μA) and voltage (6 V). The decrease in resistance along with the increase in leakage at high I_{sd}/V_{sd} are likely due to the decomposition of IL. The time-dependent resistance is constant and strongly depends on the applied current or voltage.

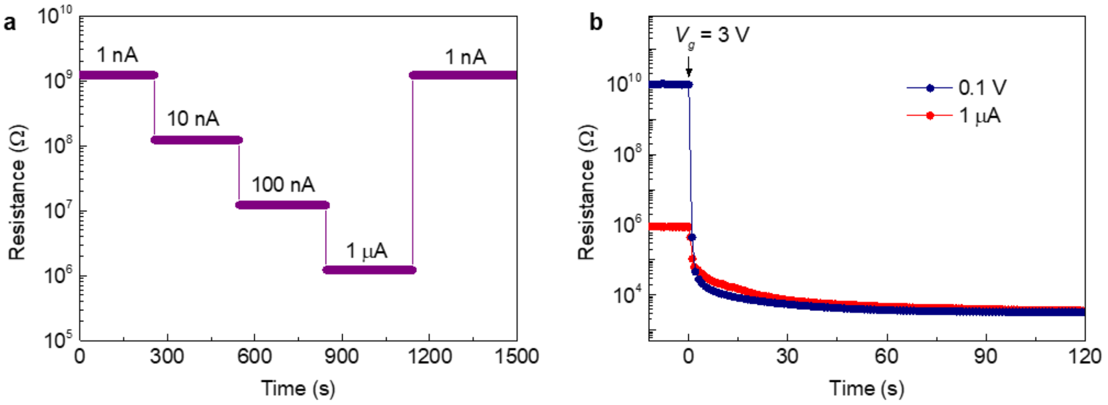

Supplementary Fig. 27. Effect of source-drain current (I_{sd}) and voltage (V_{sd}) on the resistance change of the *T*-Nb_2O_5 device. a, Time-dependent change in resistance of the *T*-Nb_2O_5 device at different I_{sd} without applying a gate voltage. I_{sd} strongly affects the resistance of device, and the resistance value is the same with the I_{sd}-dependent resistance of IL on the substrate (Supplementary Fig. 26), indicating that the observed resistance change is due to the IL, not the *T*-Nb_2O_5. Noteworthy is that the resistance change of IL is reversible even though the high current can lead to the decomposition of IL. **b**, Time-dependent resistance change of the *T*-Nb_2O_5 device at different I_{sd}/V_{sd}. The resistance decreases when gate voltage (V_g) of 3 V is applied. The initial resistance depends on I_{sd} and V_{sd}. Thus, applying low V_{sd} (0.1 V) shows larger change in resistance via metallization of *T*-Nb_2O_5.

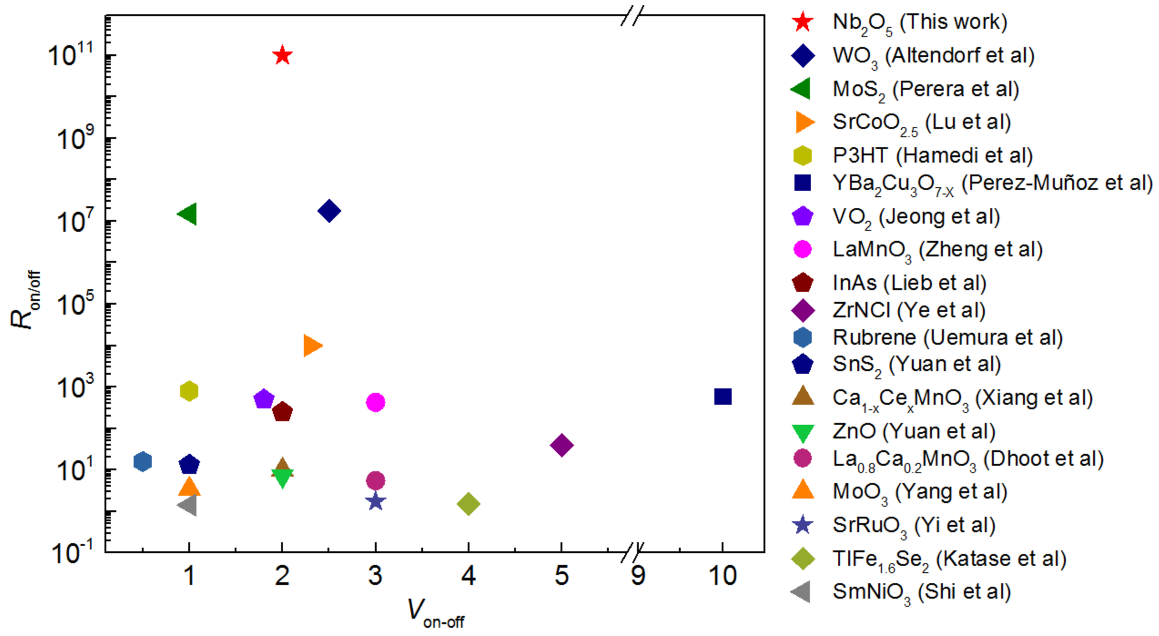

Supplementary Fig. 28. Resistance changes vs. gate voltage plot of ionic liquid gating devices. The insulating $SrTiO_3$ has shown emergent superconductivity at the low temperature after ILG, however the resistivity before gating is not presented in the reported paper[17]. Considering the high resistivity of $SrTiO_3$[18], we expect that the resistance changes of $SrTiO_3$ via ILG at ≈3.5 V will be 10^{10}–10^{11} orders of magnitude at room temperature. The detailed list are shown in Supplementary Table 1. The electrolyte gating studies using solid electrolytes are not shown here, which mostly shows slower responses and relatively small changes in resistance.

Supplementary Table 3. Resistance changes via ionic liquid gating

Material	Von-off	Ron/off	Mechanism	Migrated ion	Ionic liquid
Nb_2O_5 (This work)	2	$\approx 1\times10^{11}$	electrochemical	Lithium	Li containing DEME-TFSI
P3HT (Hamedi et al)[19]	1	$\approx 8\times10^{2}$	electrochemical	Anions	BMIM-TFSI
MoO_3 (Yang et al)[20]	1	≈3.5	electrochemical	Hydrogen	EMIM-TFSI
WO_3 (Altendorf et al)[21]	2.5	$\approx 1.8\times10^{7}$	electrochemical	Oxygen	HMIM-TFSI
$SrCoO_{2.5}$ (Lu et al)[22]	2.3	$\approx 1\times10^{4}$	electrochemical	Oxygen	DEME-TFSI
$Yba_2Cu_3O_{7-X}$ (Perez-Muñoz et al)[23]	10	$\approx 6\times10^{2}$	electrochemical	Oxygen	DEME-TFSI
VO_2 (Jeong et al)[24]	1.8	$\approx 5\times10^{2}$	electrochemical	Oxygen	HMIM-TFSI
$SrRuO_3$ (Yi et al)[25]	3	≈1.7	electrochemical	Oxygen	EMIM-TFSI
$SmNiO_3$ (Shi et al)[26]	1	≈1.4	electrochemical	Oxygen	PEMIM-TFSI
MoS_2 (Perera et al)[27]	1	$\approx 1.5\times10^{7}$	electrostatic		DEME-TFSI
$LaMnO_3$ (Zheng et al)[28]	3	$\approx 4.3\times10^{2}$	electrostatic		DEME-TFSI
InAs (Lieb et al)[29]	2	$\approx 2.2\times10^{2}$	electrostatic		EMIM-TFSI
ZrNCl (Ye et al)[30]	5	≈40	electrostatic		DEME-TFSI
Rubrene (Uemura et al)[31]	0.5	≈16	electrostatic		EMIM-TFSI
SnS_2 (Yuan et al)[32]	1	≈13	electrostatic		DEME-TFSI
$Ca_{1-x}CexMnO_3$ (Xiang et al)[33]	2	≈10	electrostatic		DEME-TFSI
ZnO (Yuan et al)[34]	2	≈7.2	electrostatic		DEME-TFSI
$La_{0.8}Ca_{0.2}MnO_3$ (Dhoot et al)[35]	3	≈5.5	electrostatic		EMIM-TFSI
$TlFe_{1.6}Se_2$ (Katase et al)[36]	4	≈1.5	electrostatic		EMIM-TFSI

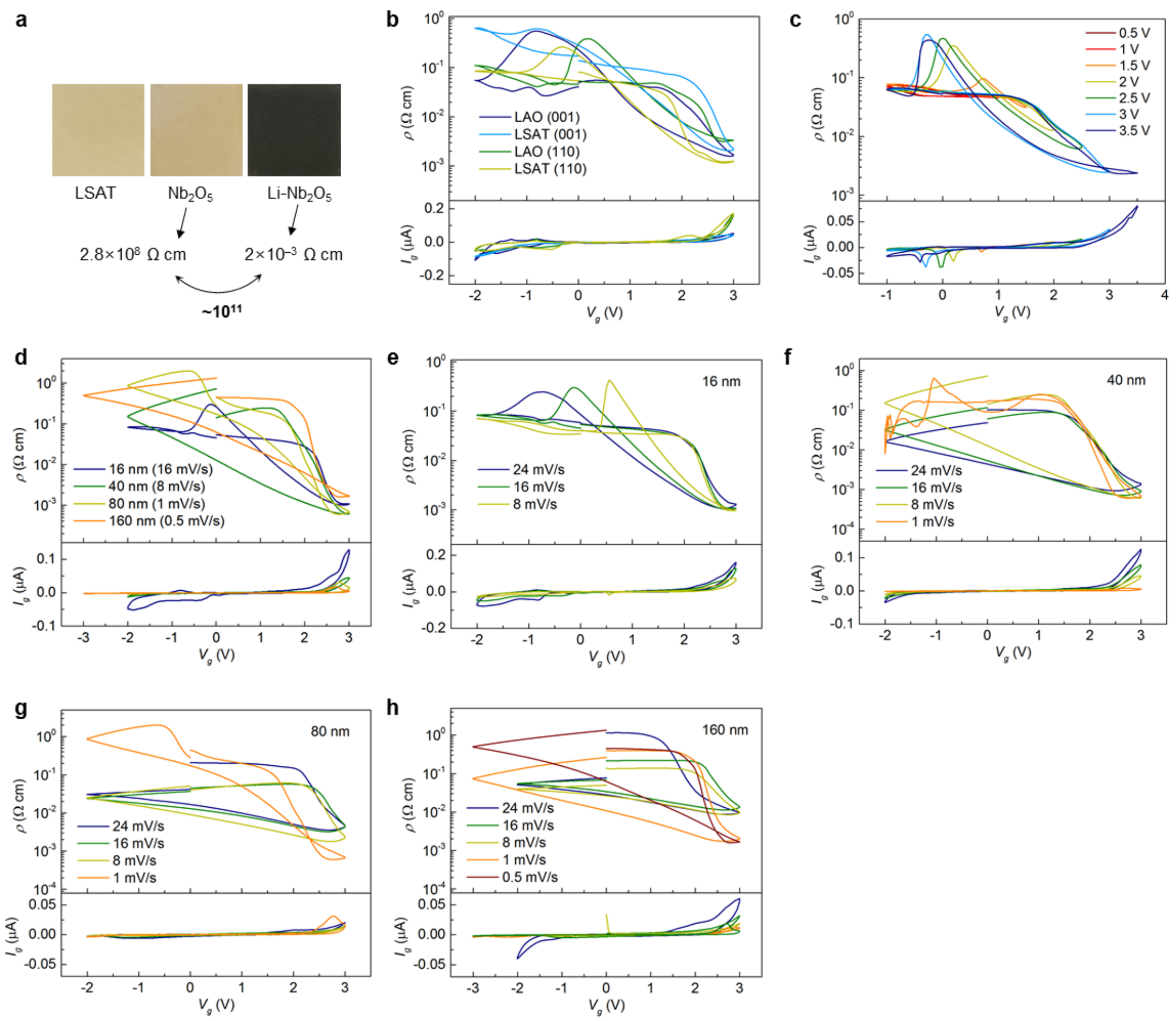

Supplementary Fig. 29. Resistance changes via ionic liquid gating for different substrate and thicknesses. a, Optical photographs of a LSAT (110) substrate, *T*-Nb_2O_5 thin films, and Li-ionic liquid gated *T*-Nb_2O_5 (Li-Nb_2O_5) thin films. The gated film shows a black color with the decrease of resistivity. The resistivity measurement was done by the Van der Pauw method at room temperature. **b**, V_g dependent resistivity (ρ) and leakage current (I_g) curves of 16 nm thick *T*-Nb_2O_5 thin film devices grown on different substrates, including LAO(001), LSAT (001), LAO (110), and LSAT (110). All devices show similar responses from gating because all films have vertically formed ionic transport channels. **c**, V_g dependent ρ and I_g curves with different maximum positive sweeping voltage for the 16 nm thick *T*-Nb_2O_5/LSAT(110) device. The resistivity decreases as increasing the maximum positive V_g. **d**, V_g dependent ρ and I_g curves with different thicknesses. V_g dependent ρ and I_g curves with different gate voltage sweeping rate for **e**, 16 nm, **f**, 40 nm, **g**, 80 nm, and **h**, 160 nm thick *T*-Nb_2O_5/LSAT(110) devices.

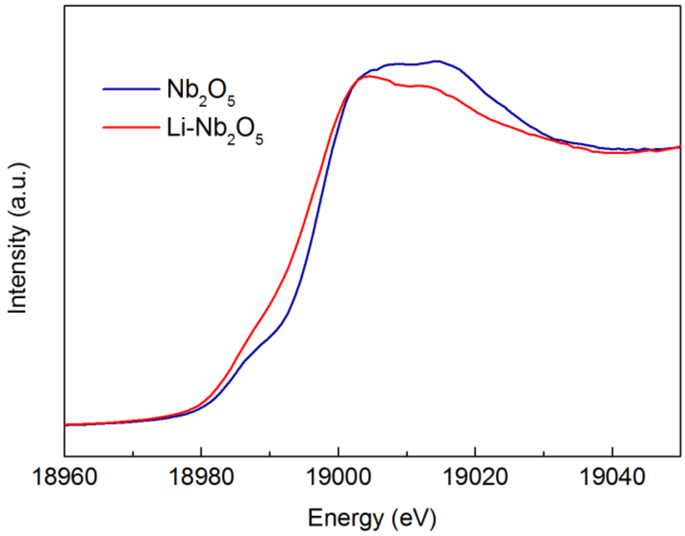

Supplementary Fig. 30. Nb K-edge XANES spectra of pristine and gated 60 nm thick *T*-Nb_2O_5 thin films. The absorption edge showed a chemical shift to a low-energy side, indicating a reduction of Nb state by Li intercalation into the lattice. The gating was done at 3 V for 20 min.

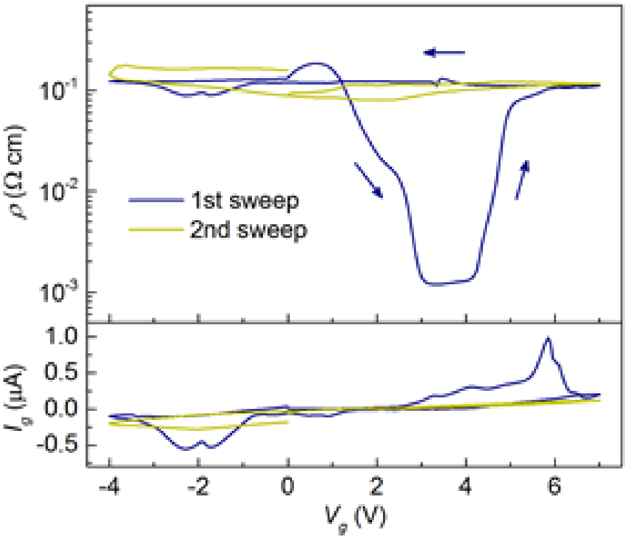

Supplementary Fig. 31. V_g dependent ρ and I_g curves with high voltages for the 16 nm thick film. The sweeping rate was 16 mV/s. The high voltage gating above ≈4.5 V lead to the insulting behavior, and the second sweeping does not show metallization, indicating the degradation of the films from the high voltage gating.

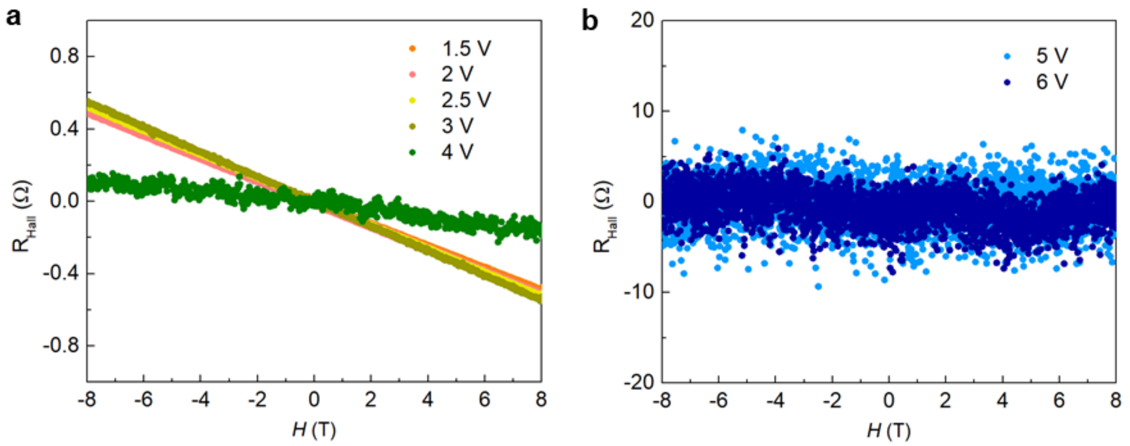

Supplementary Fig. 32. Hall measurements of the *T*-Nb_2O_5/LSAT(110) thin film device. a, Hall measurements with different gate voltages ranging from 1.5~4 V measured at 200 K. The negative signs represent that the mobile charge carriers are electrons. **b**, Hall measurements at high voltage (5 V and 6 V) lead to noisy signals due to the amorphous phase at the high voltage gating. All measurements were done for the 40 nm thick *T*-Nb_2O_5/LSAT(110) device.

6. Gate voltage tuning by using Li-containing gate electrodes

To explore the effect of the chemical potential of the gate electrode on the critical metallization voltage (V_c), the Au/Ru gate electrode is replaced by Li-containing electrode materials such as $LiFePO_4$ (LFP), $LiCoO_2$ (LCO), and $Li_xNb_2O_5$ (LNbO). The growth conditions of LFP and LCO are first optimized by PLD, and the chemical states were characterized by X-ray photoelectron spectroscopy (XPS) as shown in Supplementary Fig. 33. Then, the materials were deposited at room temperature on the Au/Ru gate electrode of the *T*-Nb_2O_5 devices (Supplementary Fig. 24b). The deposited LCO and LFP films do not show any crystalline peak from laboratory XRD measurements. In the case of the LNbO gate electrode, two *T*-Nb_2O_5 windows were first etched, and the Au/Ru electrodes and the LFPO film were deposited successively, as depicted in Supplementary Fig. 34. Then, Li ions were transferred from the LFP electrode to *T*-Nb_2O_5 gate electrode by L-ILG to form the LNbO gate electrode.

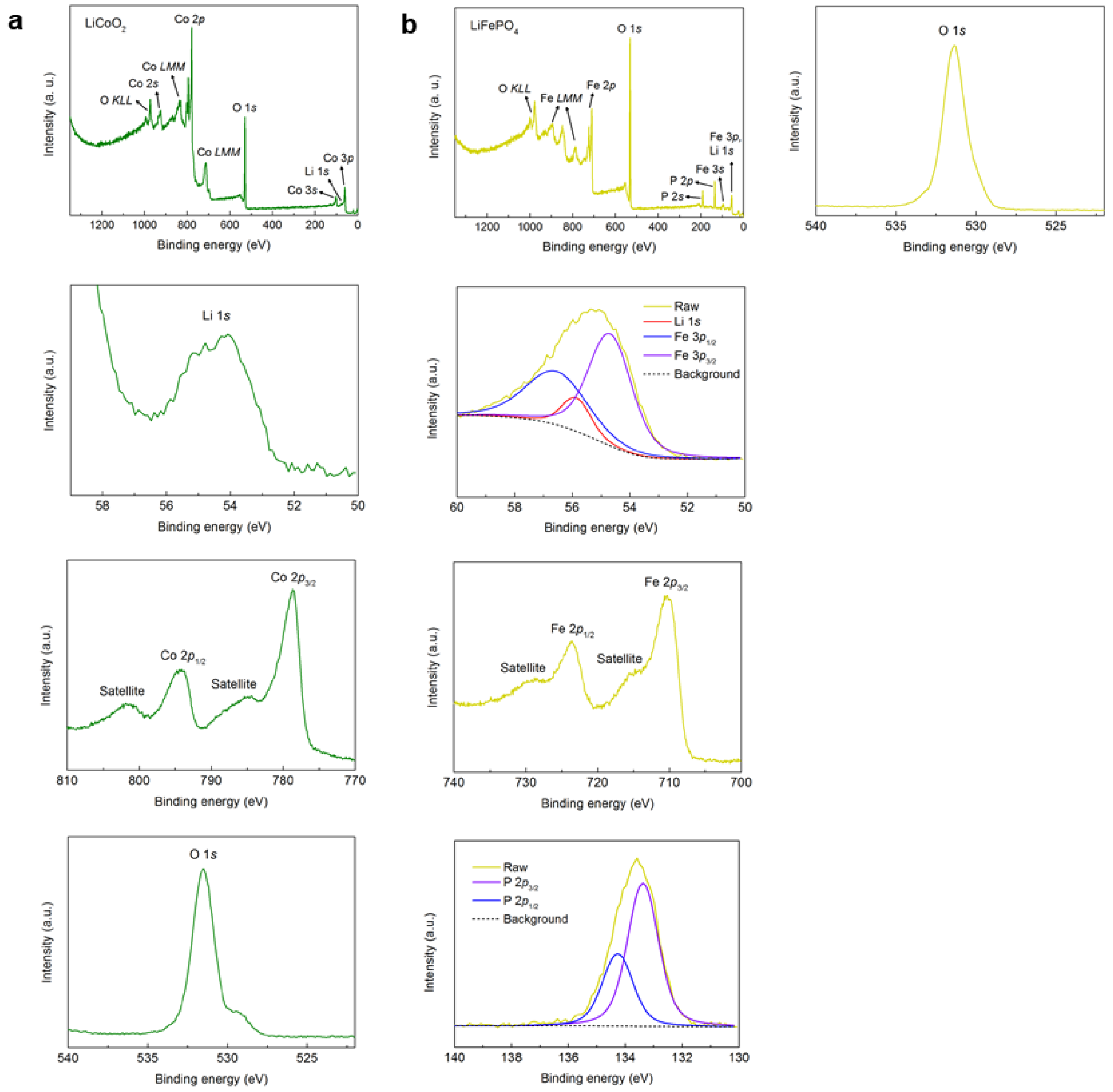

Supplementary Fig. 33. XPS results of Li-containing oxides. XPS spectra of **a**, $Li_{1+x}CoO_2$, and **b**, $Li_{1+x}FePO_4$ after growth on LSAT (110) substrates using pulsed laser deposition.

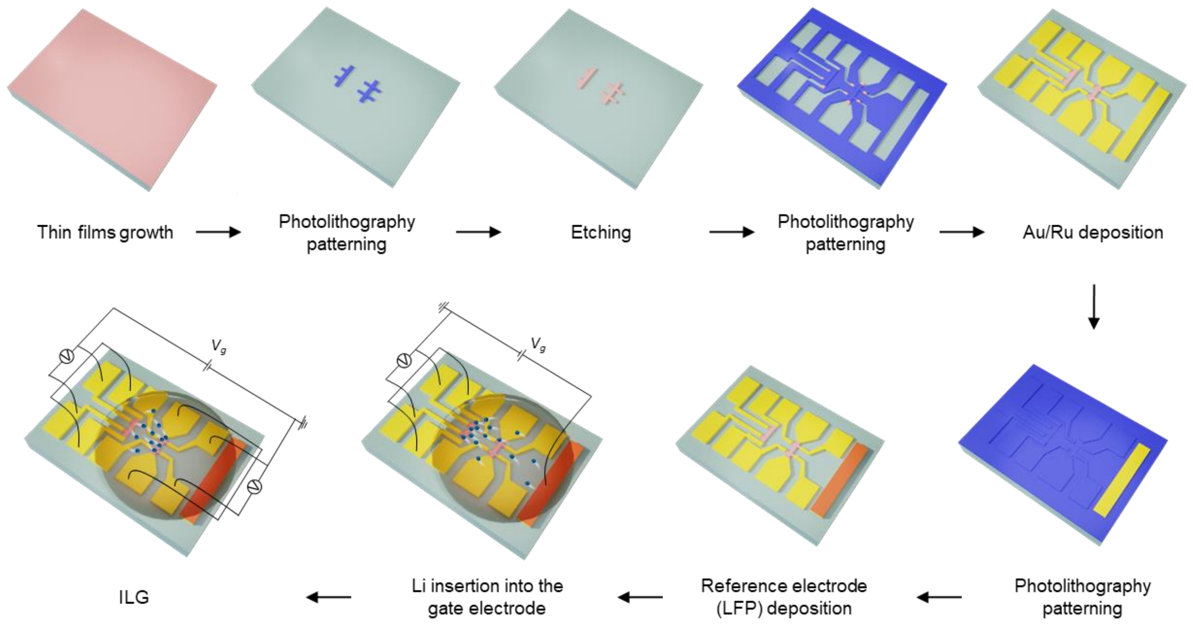

Supplementary Fig. 34. Schematic diagrams of Li-Nb_2O_5 gate electrode device fabrications. The standard photo-lithographic techniques are used. The channel and gate were etched, and then Au (70 nm)/Ru (5 nm) are deposited for the gate and channel contacts. The reference electrode (LFP) is deposited using pulsed laser deposition. After the device fabrication, Li ions are moved from the LFP to the gate electrode by ILG to make Li-Nb_2O_5 gate electrode. Then, the gating was done to the Nb_2O_5 channel using the Li-Nb_2O_5 gate electrode.

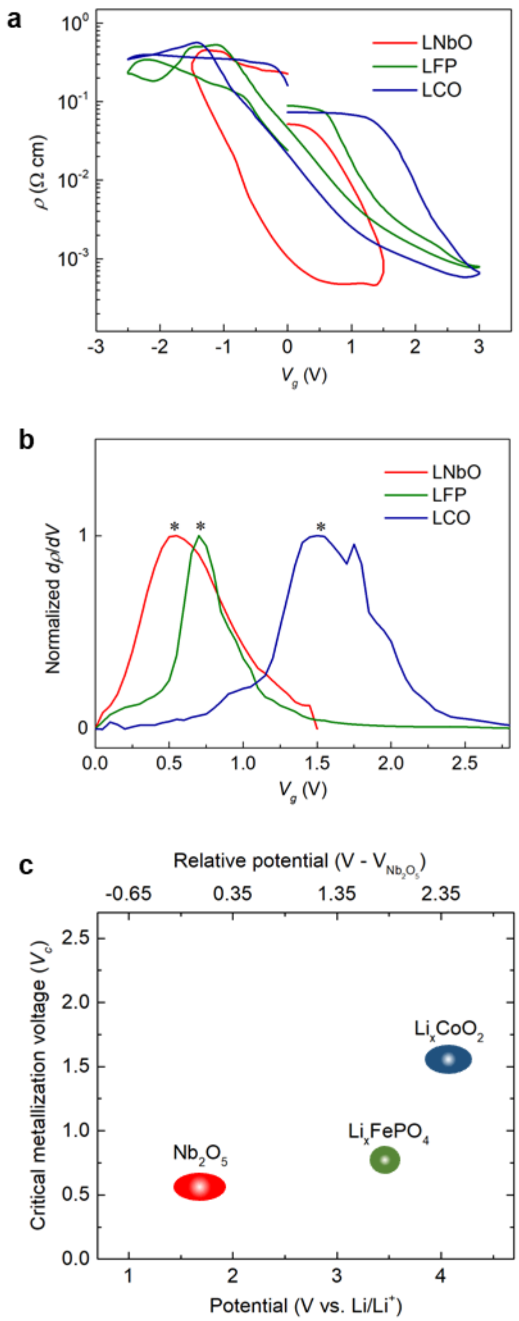

Supplementary Fig. 35. Tunable critical metallization voltage (V_c) of *T*-Nb_2O_5 thin film devices via gate electrode potential control. a, V_g dependent resistivity curves with different gate electrodes. The sweeping rate was 16 mV/s. **b**, Normalized first derivative resistive-Vg curves to determine the V_c. **c**, The chemical potential dependent V_c for different gate electrodes.

7. Electronic device performance

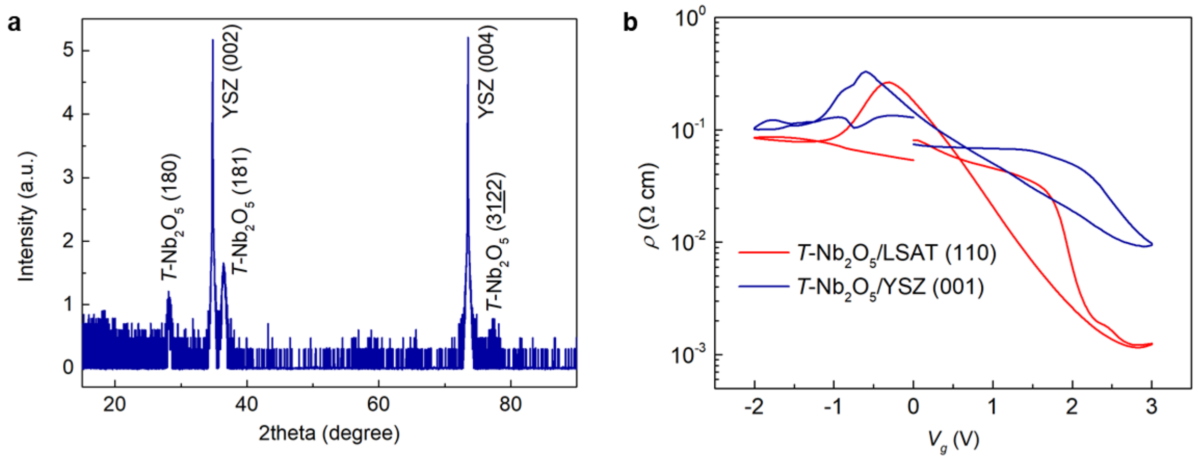

Supplementary Fig. 36. Structure and electronic properties of poly-crystalline *T*-Nb_2O_5 thin film device. **a**, Theta-2theta XRD scans of *T*-Nb_2O_5 thin films grown on a YSZ (001) substrate. The film shows multiple facets, indicating that the ionic channels are oriented in various directions. **b**, Gate voltage (Vg)-dependent resistivity curves of *T*-Nb_2O_5 thin films. The poly-crystalline *T*-Nb_2O_5/YSZ (001) shows a smaller resistance change compared to the single-crystalline *T*-Nb_2O_5/LSAT (110) having unidirectional ionic channel, for the same sweeping rate of 16 mV/sec. This represents that the vertical ionic channel orientation leads to the faster ionic migration from gating.

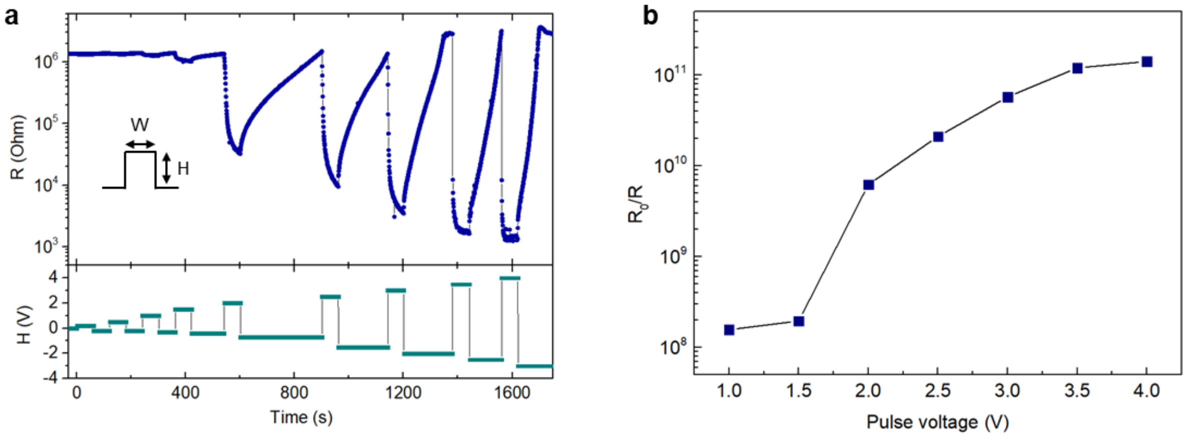

Supplementary Fig. 37. Pulsed voltage-dependent resistance change in the single crystaline *T*-Nb_2O_5 thin film device. a, Time-dependent resistance data with change of H at the fixed W = 0.5 sec. **b**, The ratio between the resistance of pristine *T*-Nb_2O_5 (R_0) and the gated resistance (R), i.e., R_0/R as a function of H, obtained from (a).

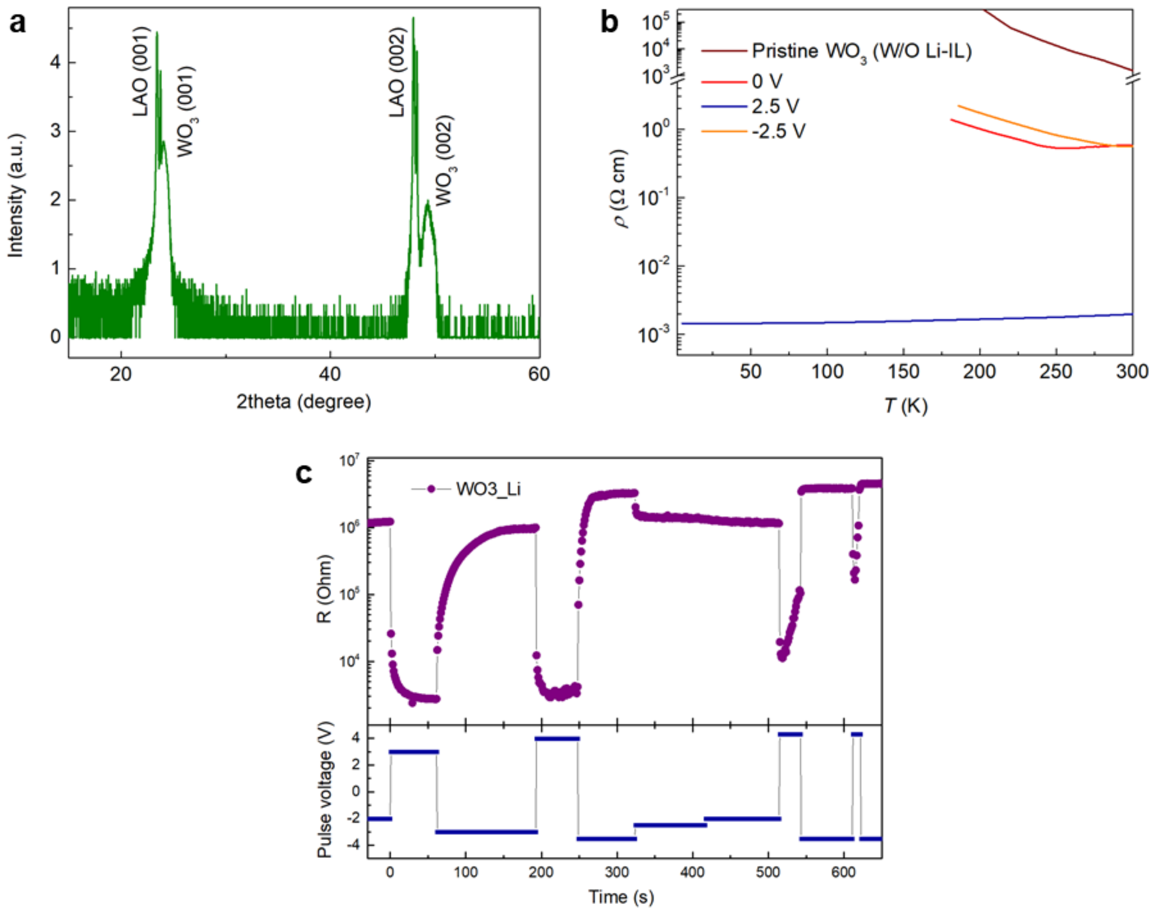

Supplementary Fig. 38. Structure and electronic properties of WO_3 thin film device. **a**, Theta-2theta XRD scans of epitaxial WO_3 thin films grown on a LAO (001) substrate. **b**, Temperature-dependent resistivity curves via Li-ILG. The resistivity of the pristine WO_3 film was measured by a high resistance meter (B2985A, KEYSIGHT). The resistivity at 0 V originates from the lower resistance of IL than the WO_3 film, as also observed for *T*-Nb_2O_5 (Fig. 2g). The film becomes metallic after gating at 2.5 V for 30 min, while it changes back to an insulating state after gating at -2.5 V for 30 min. The resistivity change from the initial film to the metallic film is ≈6 orders of magnitude. **c**, Pulse voltage gating using Li-IL. The pulse width (W) was 0.8 sec. The film becomes metallic at 3 V and 3.5 V, but the resistance rather increases when using 4~4.3 V. This could be due to the conversion reaction of WO_3 via Li intercalation.

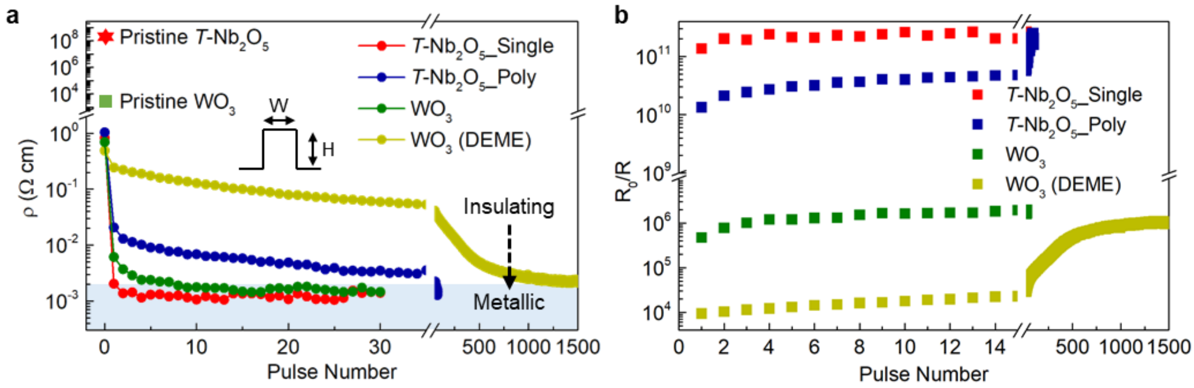

Supplementary Fig. 39. Pulsed voltage gating of single-crystalline *T*-Nb_2O_5, poly-crystalline *T*-Nb_2O_5, and WO_3 thin films. a. Resistivity changes of thin film devices as a function of number of pulses. The red, blue, and green lines denote the single-crystalline *T*-Nb_2O_5, poly-crystalline *T*-Nb_2O_5, and WO_3 thin films, respectively, gated by Li-IL. The yellow line represents the WO_3 thin film gated using DEME-TFSI. The pulse voltages (H) of 4.3, 4.3, 3.5, 4.3 V were applied for the single-crystalline *T*-Nb_2O_5, poly-crystalline *T*-Nb_2O_5, WO_3 with Li-IL, and WO_3 with DEME-TFSI, respectively. The WO_3 thin film shows a limited voltage range as it shows an increase in resistance when applying a voltage above 4 V possibly due to a conversion reaction with Li ions (See supplementary Fig. 38c). A pulse width (W) of 0.8 sec was used for all devices. The resistivities of pristine *T*-Nb_2O_5 and WO_3 are marked by a black pentacle and a square, respectively. The critical resistivity for metallization was defined as 2×10^{-3} Ω cm at room temperature, which shows a decrease in resistivity with decreasing temperature (Fig. 4f and supplementary Fig. 38b). **b**, The corresponding resistance change (R_0/R) of each film as a function of pulse numbers. R_0 represents the resistance of pristine film, while R denotes the changed resistance during gating. All film thicknesses and channel sizes were 30 nm and 60×30 μm^2, respectively.

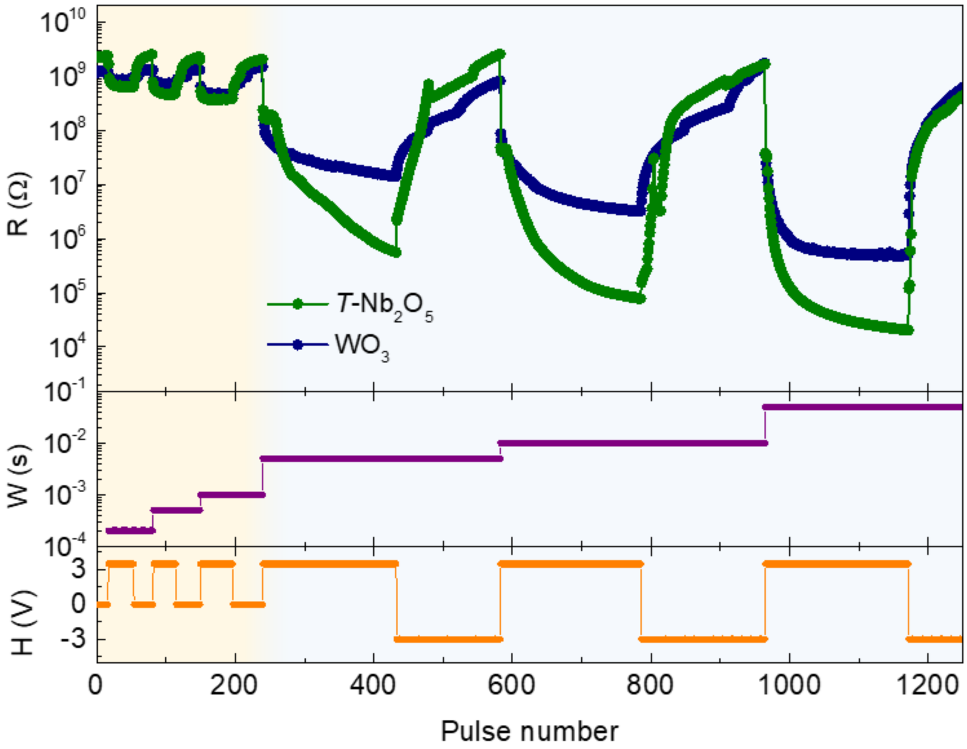

Supplementary Fig. 40. Pulse width (W)-dependent resistance changes of *T*-Nb_2O_5 and WO_3 devices. Resistance changes at different pulsed widths (W) are measured for *T*-Nb_2O_5 and WO_3 devices at V_{sd} of 0.1 V. The film thicknesses are 30 nm. The resistance of both devices do not show noticeable changes up to W of 1 ms at the gate (pulse) voltage (H) of 3.5 V, and the resistance increases back even at 0 V. This indicates that electrostatic effects are likely to be dominant at short values of W, while electrochemical intercalation into the material dominates when W is longer than 5 ms. As the electrochemical reaction via electrolyte gating occurs first via electric double layer (EDL) formation on the surface, and then subsequent ion migration into the material[37], the small change of resistance at short W (≤ 1 ms) seems to be because applied W is smaller than the EDL formation time (≈ms)[38], while the large resistance changes are observed from electrochemical intercalation into the film at longer W (5 ms) on a timescale that is longer than the EDL formation time. Notably, in the electrochemical reaction regions (above 5 ms), the resistance change of the *T*-Nb_2O_5 device is larger and faster than that of WO_3.

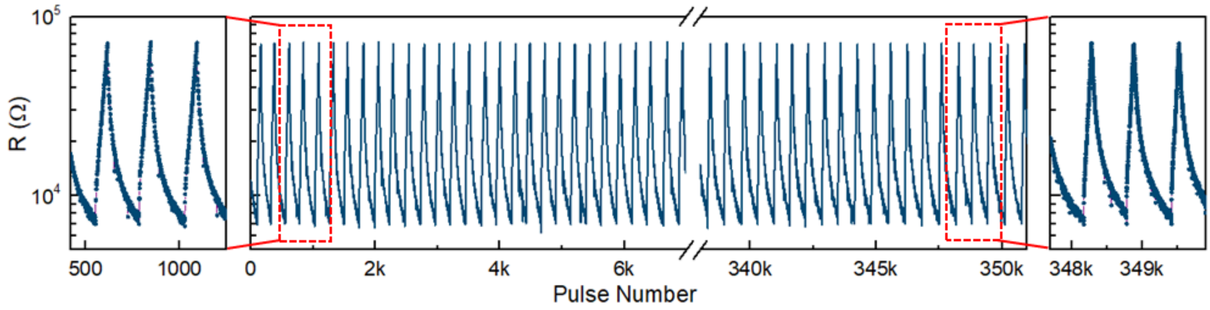

Supplementary Fig. 41. Repeatable gating of single-crystalline *T*-Nb_2O_5 thin films. Repeatable gating responses of the single-crystalline *T*-Nb_2O_5 thin film device. Pulse voltages of 3.8 V/-2 V were applied with a pulse with of 50 msec. The film thickness and channel size were 30 nm and 60×30 μm^2, respectively.

8. References in Supplementary Information.

1. Nico, C., Monteiro, T. & Graça, M. P. F. Niobium oxides and niobates physical properties: Review and prospects. *Progress in Materials Science* **80**, 1-37 (2016).
2. Giannozzi, P. *et al.* QUANTUM ESPRESSO: a modular and open-source software project for quantum simulations of materials. *J Phys Condens Matter* **21**, 395502 (2009).
3. Garrity, K. F., Bennett, J. W., Rabe, K. M. & Vanderbilt, D. Pseudopotentials for high-throughput DFT calculations. *Computational Materials Science* **81**, 446-452 (2014).
4. Perdew, J. P., Burke, K. & Ernzerhof, M. Generalized gradient approximation made simple. *Phys Rev Lett* **77**, 3865-3868 (1996)
5. Grimme, S., Antony, J., Ehrlich, S. & Krieg, H. A consistent and accurate ab initio parametrization of density functional dispersion correction (DFT-D) for the 94 elements H-Pu. *J Chem Phys* **132**, 154104 (2010).
6. Liechtenstein, A. I., Anisimov, V. V. & Zaanen, J. Density-functional theory and strong interactions: Orbital ordering in Mott-Hubbard insulators. *Phys Rev B Condens Matter* **52**, R5467-R5470 (1995).
7. Anisimov, V. V., Zaanen, J. & Andersen, O. K. Band theory and Mott insulators: Hubbard U instead of Stoner I. *Phys Rev B Condens Matter* **44**, 943-954 (1991).
8. Anisimov, V. V., Solovyev, I. I., Korotin, M. A., Czyzyk, M. T. & Sawatzky, G. A. Density-functional theory and NiO photoemission spectra. *Phys Rev B Condens Matter* **48**, 16929-16934 (1993).
9. Pinto, M. B., Soares, A. L., Jr., Mella Orellana, A., Duarte, H. A. & De Abreu, H. A. Structural, Electronic, and Thermodynamic Properties of the T and B Phases of Niobia: First-Principle Calculations. *J Phys Chem A* **121**, 2399-2409 (2017).
10. Monkhorst, H. J. & Pack, J. D. Special points for Brillouin-zone integrations. *Physical Review B* **13**, 5188-5192 (1976).
11. Henkelman, G., Uberuaga, B. P. & Jonsson, H. A climbing image nudged elastic band method for finding saddle points and minimum energy paths. *J. Chem. Phys.* **113**, 9901 (2000).
12. Henkelman, G. & Jonsson, H. Improved tangent estimate in the nudged elastic band method for finding minimum energy paths and saddle points. *J. Chem. Phys.* **113**, 9978 (2000).
13. Chen, D. *et al.* Unraveling the Nature of Anomalously Fast Energy Storage in T-Nb_2O_5. *J Am Chem Soc* **139**, 7071-7081 (2017).

14. Pinto, M. B., Soares, A. L., Jr., Mella Orellana, A., Duarte, H. A. & De Abreu, H. A. Structural, Electronic, and Thermodynamic Properties of the T and B Phases of Niobia: First-Principle Calculations. *J Phys Chem A* **121**, 2399-2409 (2017).
15. Griffith, K.J., Forse, A. C., Griffin, J. M., & Grey, C. P. High-Rate Intercalation without Nanostructuring in Metastable Nb_2O_5 Bronze Phases. *J. Am. Chem. Soc.* **138**, 8888 (2016).
16. Come, J. et al., Electrochemical kinetics of nanostructured Nb_2O_5 electrodes. *J. Electrochem. Soc.* **161**, A718 (2014).
17. Ueno, K. *et al.* Electric-field-induced superconductivity in an insulator. *Nat Mater* **7**, 855-858 (2008).
18. Wahid, M. H. A., Muhamad, N. F., Maulat Osman, R. A., Idris, M. S. & Mohd Yasin, M. N. Physical and electrical properties of $SrTiO_3$ and $SrZrO_3$. *EPJ Web of Conferences* **162**, 01052 (2017).
19. Hamedi, M. *et al.* Fiber-Embedded Electrolyte-Gated Field-Effect Transistors for e-Textiles. *Adv Mater* **21**, 573-577 (2009).
20. Yang, C. S. *et al.* A Synaptic Transistor based on Quasi-2D Molybdenum Oxide. *Advanced Materials* **29**, 1700906 (2017).
21. Altendorf, S. G. *et al.* Facet-Independent Electric-Field-Induced Volume Metallization of Tungsten Trioxide Films. *Adv Mater* **28**, 5284-5292 (2016).
22. Lu, N. *et al.* Electric-field control of tri-state phase transformation with a selective dual-ion switch. *Nature* **546**, 124-128 (2017).
23. Perez-Munoz, A. M. *et al.* In operando evidence of deoxygenation in ionic liquid gating of $YBa_2Cu_3O_{7-X}$. *Proc Natl Acad Sci U S A* **114**, 215-220 (2017).
24. Jeong, J. *et al.* Suppression of Metal-Insulator Transition in VO_2 by Electric Field-Induced Oxygen Vacancy Formation. *Science* **339**, 1402-1405 (2013).
25. Yi, H. T., Gao, B., Xie, W., Cheong, S. W. & Podzorov, V. Tuning the metal-insulator crossover and magnetism in $SrRuO_3$ by ionic gating. *Sci Rep* **4**, 6604 (2014).
26. Shi, J., Ha, S. D., Zhou, Y., Schoofs, F. & Ramanathan, S. A correlated nickelate synaptic transistor. *Nat Commun* **4**, 2676 (2013).
27. Perera, M. M. *et al.* Improved Carrier Mobility in Few-Layer MoS_2 Field-Effect Transistors with Ionic-Liquid Gating. *Acs Nano* **7**, 4449-4458 (2013).
28. Zheng, L. M. *et al.* Ambipolar ferromagnetism by electrostatic doping of a manganite. *Nat Commun* **9**, 1897, doi:10.1038/s41467-018-04233-5 (2018).
29. Lieb, J. *et al.* Ionic-Liquid Gating of InAs Nanowire-Based Field-Effect Transistors. *Advanced Functional Materials* **29**, 1804378 (2019).

30. Ye, J. T. *et al.* Liquid-gated interface superconductivity on an atomically flat film. *Nat Mater* **9**, 125-128 (2010).
31. Uemura, T. *et al.* Electronic functionalization of solid-to-liquid interfaces between organic semiconductors and ionic liquids: Realization of very high performance organic single-crystal transistors. *Applied Physics Letters* **93**, 263305 (2008).
32. Yuan, H. T. *et al.* Liquid-gated electric-double-layer transistor on layered metal dichalcogenide, SnS2. *Applied Physics Letters* **98**, 012102 (2011).
33. Xiang, P. H. *et al.* Strain-mediated phase control and electrolyte-gating of electron-doped manganites. *Adv Mater* **23**, 5822-5827 (2011).
34. Yuan, H. *et al.* High-Density Carrier Accumulation in ZnO Field-Effect Transistors Gated by Electric Double Layers of Ionic Liquids. *Advanced Functional Materials* **19**, 1046-1053 (2009).
35. Dhoot, A. S., Israel, C., Moya, X., Mathur, N. D. & Friend, R. H. Large electric field effect in electrolyte-gated manganites. *Phys Rev Lett* **102**, 136402 (2009).
36. Katase, T., Hiramatsu, H., Kamiya, T. & Hosono, H. Electric double-layer transistor using layered iron selenide Mott insulator $TlFe_{1.6}Se_2$. *Proc Natl Acad Sci U S A* **111**, 3979-3983 (2014).
37. Lück, J. & Latz, A. Modeling of the electrochemical double layer and its impact on intercalation reactions. *Phys. Chem. Chem. Phys.* **20**, 27804-27821 (2018).
38. Jänsch, T., Wallauer, J. & Roling, B. Influence of Electrode Roughness on Double Layer Formation in Ionic Liquids. *J. Phys. Chem. C* **119**, 4620−4626 (2015).
